# Designing Stimuli-Responsive Supramolecular Gels by Tuning the Non-Covalent Interactions of the Functional Groups

**DOI:** 10.3390/gels10090584

**Published:** 2024-09-11

**Authors:** Geethanjali Kuppadakkath, Ira Volkova, Krishna K. Damodaran

**Affiliations:** Department of Chemistry, Science Institute, University of Iceland, Dunhagi 3, 107 Reykjavík, Iceland

**Keywords:** LMWGs, *C*_3_-symmetric amides, amino acids, stimuli-responsive, anion sensing

## Abstract

The physical characteristics of a supramolecular gel are greatly influenced by the nature and arrangement of functional groups in the gelator. This work focuses on the impact of the functional groups, specifically the hydroxyl group, on the stimuli-responsive properties of a gel. We used a *C*_3_-symmetric benzene-1,3,5-tricarboxamide (BTA) platform, which was attached to the methyl ester of phenylalanine (MPBTA) and tyrosine (MTBTA). The gelation studies revealed that MPBTA gelled in alcohols, non-polar aromatic solvents, and aqueous mixtures (1:1, *v*/*v*) of high-polar solvents, whereas MTBTA gelled only in an aqueous mixture of DMF (1:1, *v*/*v*). The mechanical and thermal strength of the gels were evaluated by rheological and *T_gel_* studies, and the results indicated that MPBTA gels were stronger than MTBTA gels. The gels were characterized by powder X-ray diffraction and scanning electron microscopy (SEM). The analysis of stimuli-responsive properties revealed that MPBTA gels were intact in the presence of sodium/potassium salts, but the MTBTA gel network was disrupted. These results indicate that the elegant choice of functional groups could be used to tune the constructive or destructive stimuli-responsive behavior of gels. This study highlights the significant role of functional groups in modulating the stimuli-responsive properties of supramolecular gels.

## 1. Introduction

Stimuli-responsive systems are an emerging class of functional materials with versatile properties. These properties can be tuned by applying an external stimulus such as heat, light, mechanical forces, adding ions/metals, or changing the pH. Supramolecular gels based on low-molecular-weight organic molecules (LMWGs) [1,2,3,4,5] fall under this category because the gelation process can be tuned into the solution/gel phase as a function of an external stimulus [5,6,7,8,9,10]. Stimuli-responsive LMWGs are an excellent class of soft materials with a wide range of applications in the medical field as biosensors and in drug delivery, in remediation of the environment, and as self-healing materials [5,6,7,8,9,10]. The dynamic and reversible nature of these non-covalent interactions is responsible for the stimuli-responsive behavior of LMWGs [11,12]. LMWGs are formed by the self-assembly of gelator molecules, leading to the formation of one-dimensional (1-D) fibers, and these fibers entangle to form three-dimensional fibrous networks where the solvent molecules become encapsulated within these networks. Various non-covalent interactions, including hydrogen bonding, van der Waals interactions, π–π stacking, and halogen bonding, contribute to the stability of these networks. Supramolecular synthons that induce the formation of 1-D fibers, such as urea, amide, amino acids, and hydrazone moieties, have been extensively used to generate LMWGs with intriguing properties [13,14,15,16,17,18,19,20,21]. However, it is extremely challenging to predict the mechanism of the self-assembly process, as it depends on the dynamic nature of the non-bonding interactions, the nature of the functional groups, and the balance between the hydrophilic and hydrophobic interactions [1,2,3,4,5]. The nature and spatial orientation of the functional groups of the gelator have a significant impact on the physical properties of the gel, and we have shown that introducing/modifying the functional groups is an excellent strategy to generate LMWGs with tunable properties [21,22,23]. Modifying the existing gelator’s functional groups enabled the identification of the specific interactions responsible for the gel network formation. We have reported the stimuli-responsive behavior of these modified gels, such as the making and breaking of the gel network in the presence of external stimuli (salts) and their application in anion sensing [23].

Anion recognition, sensing, and binding have emerged as the pillars of supramolecular chemistry due to their broad applications in many arenas, including biology, chemistry, medicine, catalysis, and the environment [24,25,26,27,28]. Anions are ubiquitous and play a pivotal role in biological systems; for example, phosphate ions are critical for cellular energy transfer (ATP) and nucleic acid structure (DNA). The chloride ion, which is abundant in extracellular fluid, is responsible for cellular signaling, while the fluoride ion supports bone growth and prevents dental cavities [29,30,31]. Despite their vital functions, certain anions cause serious environmental and health risks. For instance, cyanide is extremely poisonous to humans [32], and phosphates commonly used in fertilizers contribute to the eutrophication of water bodies, leading to algal blooms that deplete oxygen and threaten aquatic life [33,34]. Additionally, nitrate and sulfate are key components in forming acid rain, which can harm ecosystems and infrastructure [35]. Thus, anion sensing is important in medical diagnostics and for the detection of deleterious anions in the environment. The stimuli-responsive properties of the material in the presence of anions can be utilized to detect the presence of the anionic species [36]. The presence of supramolecular synthons in LMWGs that could interact with the anions makes them ideal candidates for anion-sensing application, and the stimuli-responsive behavior can be monitored by the sol–gel transitions.

There are three primary approaches for anion sensing with a gelator molecule. First, gelators may undergo direct chemical reactions with the anion, in which specific reactive sites within the gelator molecule react with the introduced anion [37]. Second, the anion can disrupt the gel network of a metal ion or cation incorporated supramolecular gel network by electrostatic interactions [6]. Finally, anion sensing can occur via hydrogen-bonding interactions [6] because most LMWGs contain supramolecular synthons [38], and these functional groups interact with the anions through non-covalent interactions. In this approach, the hydrogen-bonding functionalities of the gelator interact with the anions, leading to constructive or destructive interference, which may result in anion-induced gelation or gel network disruption. LMWGs with hydrogen-bonding functional groups urea, amide, hydroxyl, sulphanilamide, arylhydrazone, and nucleoside have been employed for anion sensing [23,37]. Among these, amide moieties are particularly notable [6,18,22,39] due to their complementary N–H···O=C interactions, where the donor (N-H) and acceptor (C=O) of the amide units facilitate the formation of 1-D fibril structures. The donor and acceptor moieties of the amide units can readily interact with the anions [40], and we have shown that amide-based LMWGs are excellent candidates for anions-sensing applications [21,22]. The nature and spatial orientation of functional groups in a gelator could interfere with the binding modes of the anions, which may have a significant impact on the anion-sensing properties of the LMWGs [41]. In this work, we investigate the role of the functional group on the anion sensing capabilities of the LMWGs gels, specifically the hydroxyl group by comparing the gelation properties of *C*_3_-symmetric trimesic amide of the methyl ester of phenylalanine (MPBTA) [42] and benzene-1,3,5-tricarboxamide of the methyl ester of tyrosine (MTBTA), with an additional hydroxy group. LMWGs based on *C*_3_-symmetric trimesic amide are reported for anion sensing [6,43,44,45,46,47,48], but the combination of functional groups such as amide and amino acid ester moieties in *C*_3_-symmetric-trimesic-amide-based LMWGs as an anion sensor are not known.

## 2. Results and Discussion

### 2.1. Synthesis of MPBTA and MTBTA

**Scheme 1:**
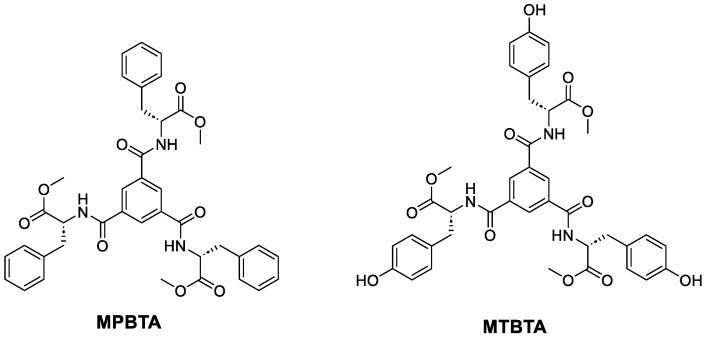
Chemical structure of MPBTA and MTBTA.

The *C*_3_-symmetric trimesic (benzene-1,3,5-tricarboxamide, BTA) family of compounds are well-explored supramolecular platforms, better known for their ease of synthesis, flexibility towards structural modification, and the tendency to form nanofibers that facilitate gelation in a wide range of solvents [49,50]. We have shown that combining functional groups such as the amide and amino acid ester moieties in a single gelator offers a better understanding of structure–property correlations of LMWGs [39]. In this work, we selected the trimesic amides of the methyl esters of phenylalanine and tyrosine (Scheme 1), which comprise various functionalities such as the central aromatic platform, dangling aromatic groups, polar amino acid ester moieties, and large non-polar groups to analyze the role of functional groups on the anion binding abilities of LMWGs. The *R*,*R*,*R*-benzene-1,3,5-tricarbonyl phenylalanine methyl ester (MPBTA, Scheme S1) was synthesized by reacting the corresponding enantiopure (*R*) methyl ester hydrochloride of phenylalanine with benzene-1,3,5-tricarbonyl trichloride in dry dichloromethane [42]; while, the *R*,*R*,*R*-benzene-1,3,5-tricarbonyl tyrosine methyl ester (MTBTA) was synthesized using the silyl-protected enantiopure (*R*) methyl ester hydrochloride of tyrosine (Scheme S1). The enantiopurity of MPBTA and MTBTA were studied in absolute ethanol using solution-state circular dichroism (CD) experiments (see experimental section for details).

### 2.2. Gelation Studies

A standardized protocol was used to perform the solvent screening to determine the appropriate solvents/solvent mixtures for the MPBTA and MTBTA gelation experiments. The compound (10.0 mg) was accurately weighed and added to a standard vial (7.0 mL), 1.0 mL of the selected solvent was added, and the vial was sealed. The mixture was then subjected to sonication and heating until a clear solution was achieved. For the aqueous mixtures of polar organic solvents (1:1, *v*/*v*), the compound was dissolved through sonication and heating in the organic solvent, and water was added. The resulting mixture underwent sonication and a heating process to redissolve the gelator. The vials were inverted after 24 h to check the gel formation. The results showed that MPBTA gelled in alcohols such as methanol, ethanol, *n*-propanol, isopropanol, *n*-butanol, 2-butanol, and *n*-pentanol and in non-polar aromatic solvents such as xylenes and mesitylene (Table S1). Gelation was unsuccessful in aliphatic solvents, presumably due to their high solubility. However, the gelation experiments performed with MTBTA, which had additional hydroxy functionalities, did not form gels with these solvents. MTBTA was insoluble in apolar aromatic solvents and formed a clear solution in other solvents (Table S1). The gelation ability of MPBTA and MTBTA compounds was evaluated in the aqueous mixtures (1:1, *v*/*v*) of polar protic and aprotic solvents. Gelation was observed for MPBTA in the aqueous mixtures of methanol, ethanol, DMF, and DMSO (Table S1). The gelation test of MTBTA revealed that gel formation was observed only in an aqueous mixture of DMF (1:1, *v*/*v*). Crystallization was observed in the aqueous mixtures (1:1, *v*/*v*) of methanol and ethanol, but a partial gel was observed for DMSO/water (1:1, *v*/*v*) at higher concentrations (8.0 wt/v%). This unique behavior may be attributed to the hydroxyl group in MTBTA, which interferes with the formation of 1-D hydrogen-bonded networks, thereby inhibiting gel formation in other solvents. The hydroxyl group may increase the hydrophilicity of MTBTA, making it more soluble in polar solvents but less soluble in apolar xylenes and mesitylene (Table S1).

The minimum gelator concentration (MGC), which denotes the least amount of gelator required to establish a rigid gel network in the given solvent, was determined by altering the gelator composition (Table 1 and Table S2). The MGC of MPBTA was lower in *p*-xylene compared to other xylenes and mesitylene, and for alcohols, increasing the carbon chain decreases the MGC, presumably due to the lower solubility of MPBTA in higher-aliphatic alcohols. Analyzing the MGC in the aqueous mixtures (1:1, *v*/*v*) revealed that MPBTA forms a gel at a concentration below 1.0 wt/v% in DMSO/H_2_O and EtOH/H_2_O, which can be considered as a super gelator in these solvents. The comparison of the MGC of MPBTA and MTBTA indicated that the MGC of MTBTA in (1:1, *v*/*v*) was higher (4.5 wt/v%) than that of MPBTA (1.4 wt/v%) (Table 1). The hydrophilicity and potential for disruptive hydrogen bonding associated with the additional hydroxyl group in MTBTA might have affected the gelation process, leading to a higher MGC than that of MPBTA.

### 2.3. Thermal Stability

The thermal stability of MPBTA and MTBTA gels was compared by evaluating the sol–gel phase conversion temperature (*T_gel_*) at which the gel shifts to the liquid phase. Gels were prepared at identical concentrations to ensure that their stability was compared under similar conditions. The *T_gel_* value of MTBTA gel was lower than that of MPBTA gel in DMF/water, indicating a weaker gel network. MTBTA was more soluble in the DMF/water system compared to MPBTA, and the lower solubility of MPBTA may allow for stronger interactions between fibers in the gel, which in turn allows for higher thermal stability (Table 1). MPBTA gel in *p*-xylene exhibited greater thermal stability than other polar solvent gels. This enhanced stability was probably due to the better gel network formation of MPBTA molecules in *p*-xylene facilitated by the presence of four hydrophobic benzene rings in the gelator, which could interact with the aromatic ring of *p*-xylene via π–π interactions.

### 2.4. Rheology

**Figure 1:**
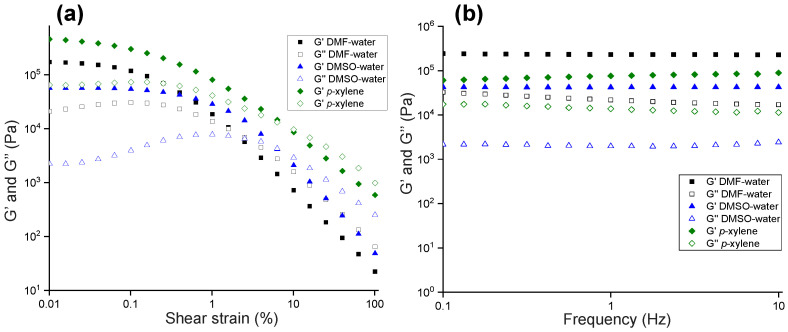
Rheological experiments were performed on MPBTA gels at 6.5 wt/v% in the aqueous mixtures (1:1, *v*/*v*) of DMF and DMSO, and in *p*-xylene at 20.0 °C with a constant strain of 0.01%: (**a**) amplitude-sweep and, (**b**) frequency-sweep experiments.

Rheology is an essential technique for probing the stiffness, fluid properties, and deformation of gels, yielding a profound overview of the gel network’s structural aspects [51,52]. The mechanical strength of the MPBTA gel was analyzed by executing amplitude and frequency-sweep experiments in *p*-xylene and aqueous mixtures (1:1, *v*/*v*) of DMF and DMSO at 6.5 wt/v%. Amplitude sweep measurements were conducted at a constant frequency of 1.0 Hz to identify the linear viscoelastic region (LVR) of the gels, characterized by reversible deformation and preservation of the initial structure. The gels were prepared in the corresponding solvents, and the rheological experiments were carried out after one day by scooping the sample onto the Peltier plate of the rheometer. The results showed that all the gels displayed a constant G′ up to 0.015% strain (Figure 1a).

**Figure 2:**
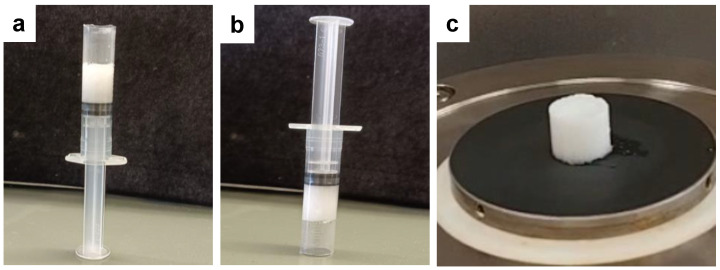
An illustration of the syringe method used to prepare gels, (**a**) gel formation within the syringe; (**b**) pressing the syringe piston (**c**) gel on the rheometer.

Frequency-sweep experiments were conducted within the LVR region with a constant strain of 0.01% across a frequency range of 0.1–10.0 Hz, and results indicated that MPBTA gels in DMF/water exhibited a higher mechanical strength compared to the gels in *p*-xylene and DMSO/water (Figure 1b). To compare the mechanical strength of MPBTA and MTBTA, we used a syringe method to prepare the gel instead of the scooping method due to the soft nature of the MTBTA gel in DMF/water (1:1, *v*/*v*). This was performed by chopping off the syringe tip, and the hot gelator solution (6.5 wt/v%) was poured into the syringe body. The resulting gel was left to age overnight (Figure 2a). The rheology was performed by transferring the gels onto the rheometer by pressing the syringe piston (Figure 2b,c). Then, the amplitude-sweep experiments were performed (Figure S1).

**Figure 3:**
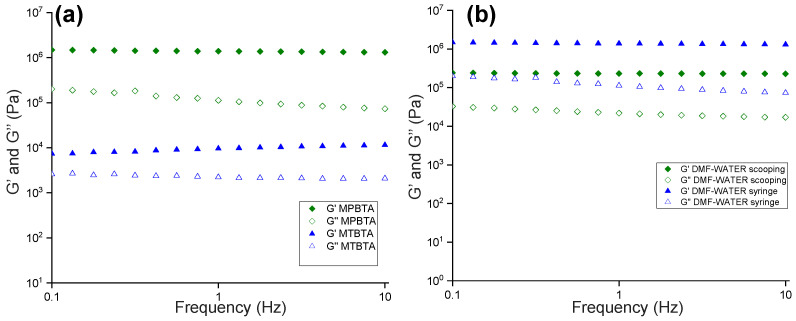
Frequency-sweep experiments performed at 20.0 °C with a constant strain of 0.01%; (**a**) comparison of the mechanical strength of MPBTA and MTBTA in an aqueous mixture (1:1, *v*/*v*) of DMF at 6.5 wt/v% and, (**b**) comparison of the mechanical strength obtained from scooping and syringe methods.

A comparison of the G′ values of MPBTA and MTBTA gels in DMF/water suggested that MPBTA gel was stiffer than MTBTA gel (Figure 3a). This is presumably due to the additional hydroxyl group in MTBTA, which disrupts the complementary hydrogen bonding, leading to weaker gels and consequently lowering the thermal and mechanical stability. 

We also compared the mechanical strength of MPBTA gels obtained from the scooping method and syringe method to check whether the mechanical strength depends on the sample preparation process, and the results indicated similar trends in DMF/water (Figure 3b) and DMSO/water (Figure S2). The same method was applied for MPBTA gels in *p*-xylene, but the *p*-xylene-based mixture gelled too quickly, even after extensive heating, and gelled on the sides of the syringe (Figure S3), preventing a uniform gel formation within the syringe.

### 2.5. Gel Morphology

**Figure 4:**
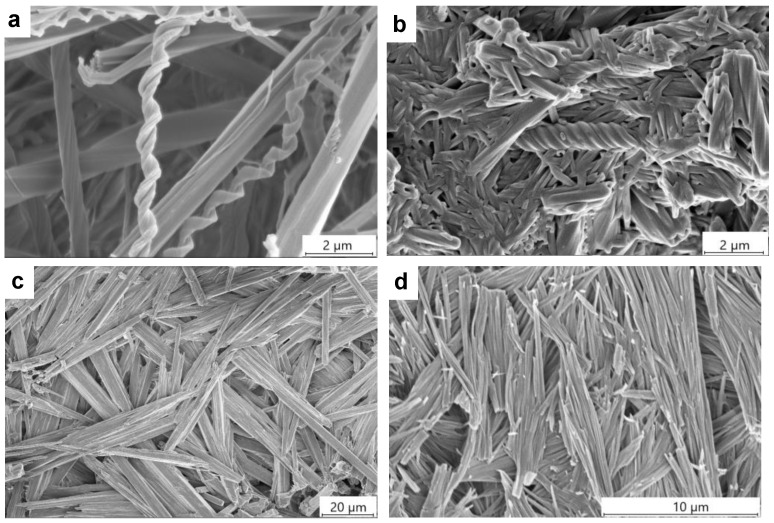
SEM images of xerogels (**a**) MPBTA gels from *p*-xylene (1.0 wt/v%); (**b**) DMSO/water (1:1, *v*/*v*, 3.0 wt/v%); and xerogels of MPBTA (**c**) and MTBTA (**d**) from DMF/water (1:1, *v*/*v*) at 6.5 wt/v%, respectively.

The surface morphologies of the MPBTA and MTBTA gels were analyzed by scanning electron microscopy (SEM), which is a powerful tool for visualizing the microstructure of gels [11]. SEM was performed on the xerogels of MPBTA and MTBTA, obtained by filtering the gels after 24.0 h and air-drying the remaining material in the fume hood. Although the morphologies of the dried gels structurally represent the actual gel networks, the drying may introduce some artifacts leading to different morphologies in some cases [53]. The dried gels of MPBTA were prepared from *p*-xylene (1.0 wt/v%), *m*-xylene (3.0 wt/v%), and DMSO/water (1:1, *v*/*v*) at 3.0 wt/v%. We also prepared the dried gels of MPBTA and MTBTA in DMF/water (6.5 wt/v%) for comparison. The SEM images of the MPBTA xerogel from *p*-xylene (Figure 4a) and DMSO/water (Figure 4b) displayed similar morphologies with helical fibers and tape-shaped morphologies, and the thickness ranged from 0.1–2.0 µm. The helical fibers showed counterclockwise twisting, indicating the preservation of chirality in the gel fibers and similar morphologies were observed for the *m*-xylene-based xerogel (Figure S4).

The SEM images of MPBTA and MTBTA from DMF/water (6.5 wt/v%) were compared to analyze the role of the additional hydroxy group, and the fibers displayed a needle-shaped morphology, indicating a minor difference in the morphologies (Figure 4c,d). However, further analysis revealed that the MTBTA xerogels displayed a slightly twisted needle-like morphology with thickness ranging from 0.1 to 1.0 μm, but thicker fibers (1.0–5.0 μm) with a straight needle-like morphology were observed for the MPBTA gels.

### 2.6. Single-Crystal X-ray Diffraction

**Figure 5:**
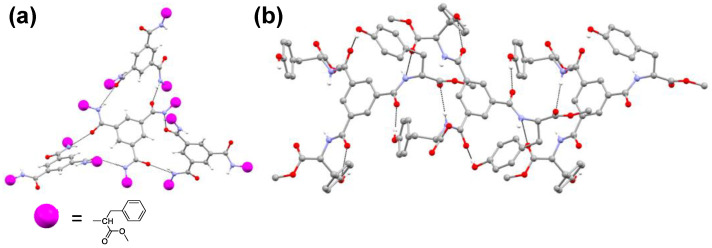
Crystal structures of MPBTA [54] reproduced with permission from Elsevier and MTBTA; (**a**) the complementary hydrogen-bonding interactions (dotted lines) between the N-H and C=O moieties of the amide groups in MPBTA and (**b**) Two types of interactions observed in MTBTA, where the hydroxy group interacts with the amide carbonyl moiety and the N-H moiety is hydrogen bonded to the carbonyl moiety of the two ester groups, color code: C-atom (grey), nitrogen atom (blue) and oxygen (red).

The crystallization experiments with MPBTA and MTBTA were performed in methanol, ethanol, and the aqueous mixtures of methanol and ethanol (2:1, *v*/*v*), respectively. Single crystals of MPBTA suitable for X-ray analysis were obtained from ethanol, while MTBTA formed crystals in ethanol/water (2:1, *v*/*v*). Single-crystal X-ray diffraction (SCXRD) was conducted on MPBTA and MTBTA crystals to elucidate the solid-state structural details of the gelator molecules, which helps to correlate the non-covalent interactions that drive the self-assembly process in the gel state. The unit cell dimensions of MPBTA matched with the reported structure [54]. MTBTA was crystallized in a monoclinic *P*2_1_ space group, and the unit cell parameters (Table S3) matched with the enantiomeric (*S*,*S*,*S*)-MTBTA [55]. We compared the crystal structures of MPBTA and MTBTA to evaluate the mode of interactions, and the results indicated the presence of complementary hydrogen bonding interactions in MPBTA between the N-H and C=O moieties of the amide groups (Figure 5a), resulting in a 2-D hydrogen-bonded network. These networks were further stabilized by a face-to-face π-π interaction between the center benzene ring and the aromatic moieties of the phenylalanine [52]. However, in MTBTA, the additional hydroxy group disrupted the complementary hydrogen bonding interactions between the amide moieties. The hydroxy group was hydrogen bonded to the oxygen atom of the amide carbonyl moiety and the N-H moiety displayed hydrogen bonds with the carbonyl moieties of the two ester groups (Figure 5b) leading to a 3-D hydrogen-bonded network. One of the ester moieties was not involved in this hydrogen bonding but displayed non-bonding interactions with the adjacent molecules. These results indicate that the mode of the non-bonding interactions can be tuned/disrupted by adding additional functional groups.

### 2.7. Powder X-ray Powder Diffraction (PXRD)

**Figure 6:**
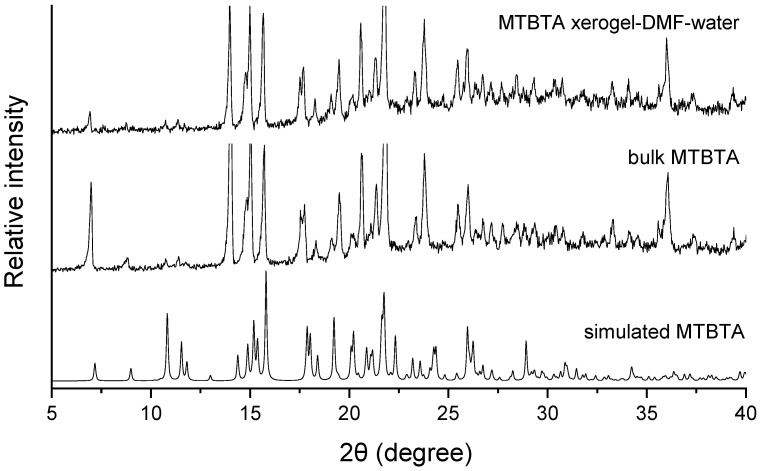
Comparison of PXRD patterns of MTBTA xerogels from DMF/water (1:1, *v*/*v*) at 5.0 wt/v%, bulk crystals and simulated pattern of single crystal.

Powder X-ray diffraction (PXRD) is a useful analytical technique that provides valuable information on phase purity and molecular packing [11,17]. This technique is particularly useful for LMWGs, as it compares the simulated pattern generated from single-crystal data with the PXRD pattern of bulk materials or xerogels, which offers a better understanding of self-assembly modes within the gel matrix [22,50]. However, this method has some limitations due to potential artifacts introduced during the preparation of xerogels in some cases [53]. We performed PXRD experiments on the bulk crystals of MPBTA and MTBTA, and the patterns were compared with the simulated patterns of the corresponding crystal structure to validate the phase purity. The bulk and simulated patterns were superimposable for MTBTA (Figure 6) and similar results were observed for MPBTA (Figure S5). 

The gels of MPBTA (5.0 wt/v%) obtained from *p*-xylene, DMF/water (1:1, *v*/*v*), and DMSO/water (1:1, *v*/*v*) were filtered and dried, and PXRD experiments were performed on these xerogels (Figure S6). The PXRD pattern of the xerogels indicated superimposable patterns, with most peaks matching the simulated pattern, indicating a similar gel network in all solvents (Figure S6), but the PXRD pattern obtained from DMSO/water xerogel showed broad peaks, probably due to the weakly diffracting gelator’s microcrystal. The PXRD pattern of the MTBTA xerogel derived from DMF/water (1:1, *v*/*v*) at 5.0 wt/v% perfectly aligned with the simulated pattern of MTBTA (Figure 6). These results indicate that the mode of interactions and the crystal packing of the xerogels of MPBTA and MTBTA were similar to their corresponding crystal structures.

### 2.8. Infrared Spectroscopy

Attenuated total reflectance–Fourier transform infrared (ATR–FTIR) spectroscopy is an excellent technique for unraveling the hydrogen-bonding interactions in supramolecular gels [11,56,57,58,59], which can be achieved by comparing the solid-state stretching and bending peaks of the functional groups with the corresponding peaks in the xerogel/gel state. The shift in the IR peaks can be corroborated to the extent of hydrogen-bonding interactions in the supramolecular gels. The N-H stretching peaks provide insight into the degree of hydrogen bonding between amide groups, and comparing these peaks in the solid state and the gel state can reveal the structural details of the self-assembly and molecular interactions within the gel network. The ATR-FTIR spectra of MPBTA (2.0 wt/v%) and MTBTA (4.5 wt/v%) in DMF/D_2_O (1:1, *v*/*v*) were recorded in the gel state and compared with the IR spectra of the crystals and xerogels (Figures S7–S10 and Table S4). The background spectrum of the solvents in the gel state was recorded using DMF/D_2_O (1:1, *v*/*v*). The spectra showed that N―H stretching vibration signals of MPBTA appeared between 3025 cm^−1^ and 3402 cm^−1^ and from 2936 cm^−1^ to 3228 cm^−1^ for MTBTA. Analysis of the peaks revealed that in the gel state, N―H stretching peaks were shifted towards lower wavenumbers, indicating an extended hydrogen bonding (Table S4). Similarly, the O–H stretching peak of the phenol group in MTBTA was shifted towards a lower wavenumber in the gel state and xerogel (Table S4). There was no drastic shift in the C=O stretching peaks of the amide and the ester moieties in MPBTA, which corroborated well with the crystal structure (Table S4). However, in MTBTA crystals, the C=O stretching peaks of ester moieties were split into two peaks (1718 and 1755 cm^−1^), indicating the presence of two types of interactions. This was compared with the crystal structure of MTBTA, which confirmed the presence of two types of non-bonding interactions in the ester moieties. The ester moieties interacting with the N-H moieties were shifted in the xerogel and the gels state, but the shift was nominal for the other ester group. The C=O stretching peaks of amide in MTBTA crystals were shifted to the lower values in the xerogel and gel state (Table S4). The shifts in the IR peaks may be attributed to the extensive hydrogen bonding of the functional groups arising from the self-assembled gel network.

### 2.9. Stimuli-Responsive Properties 

**Figure 7:**
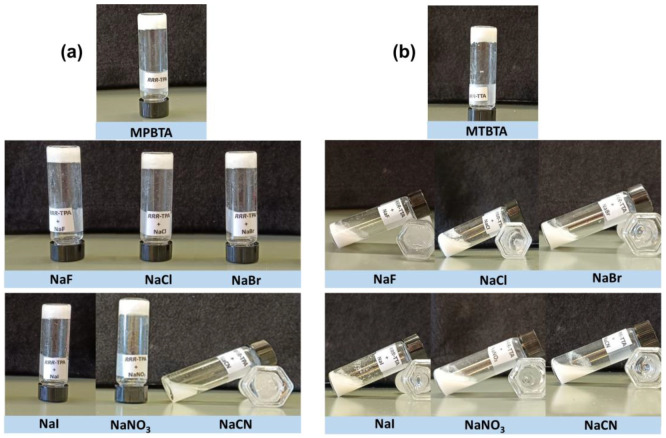
Stimuli-responsive properties of the (**a**) MPBTA gels (1.4 wt/v%) and (**b**) MTBTA gels (4.5 wt/v%) in DMF/water mixture (1:1, *v*/*v*) towards various sodium salts.

The stimuli-responsive characteristics of MPBTA gels towards anions were investigated by exposing them to different anions (halides, nitrate, and cyanide) of sodium and potassium salts. Initially, we analyzed the effect of anions at MGC (1.4 wt/v%) by dissolving 14.0 mg of MPBTA in DMF (0.5 mL), followed by the addition of an aqueous solution (0.5 mL) of the given anion (1.0 equiv.). The mixture was then heated and sonicated to redissolve the gelator and left untouched for 24 h. The results indicated that the gel network was stable in the presence of halides and nitrate ions, but cyanide salts disrupted the gel network for sodium (Figure 7a) and potassium (Figure S11) salts. 

The thermal and mechanical strength of the MPBTA gels with anions were characterized by *T_gel_* and rheological experiments, which were performed to assess their strength compared to the original gel. The results indicated that the mechanical and thermal stability slightly increased in the presence of anions (Figure S12 and Table S5). Subsequently, we attempted the gelation below the minimum gelator concentration (1.4 wt/v%) with these anions (halides and nitrate) to explore the potential of anion-induced gelation, and anion-triggered gelation was observed in all cases. The MGC for anion-induced gelation was found to be 0.9 wt/v% for MPBTA. The impact of anions on the stability of the gel networks was studied in detail by treating MPBTA gels with 1.0 and 3.0 equivalents of sodium/potassium (1.0 equiv.) halides and nitrate ions at 1.8 wt/v% (above MGC) in DMF/water mixture (1:1, *v*/*v*). After one day, the rheological experiments performed on these gels revealed enhanced mechanical strength in the presence of 1.0 equivalents of anion compared to the native gel at identical concentrations (Figures S13 and S14). However, the mechanical strength decreased in all cases except for the gel with fluoride ion as the concentration of anion increased.

Similarly, the anion-responsive behavior of MTBTA gels was investigated in DMF/water (1:1, *v*/*v*) at MGC (4.5 wt/v%) in the presence of the anions (1.0 equiv.) using the same procedure as for MPBTA. The results showed that the gel networks were disrupted by the presence of anions of sodium (Figure 7b) and potassium (Figure S15) salts in all cases. The experiments performed at higher MTBTA concentration (5.0 wt/v%, above MGC) resulted in partial gels, indicating that the anions show destructive interactions with the MTBTA gel networks. The experiments were repeated with multi-valent sulfate and phosphate anions such as sodium/potassium sulfate and sodium pyrophosphate salts (Table S6). The MPBTA gels remained intact, but the MTBTA gel network collapsed in the presence of multi-valent anions, indicating a similar trend as the mono-valent anions. (Figure S16). We analyzed the mechanical strength of the MPBTA gels in the presence of 1.0 and 3.0 equivalents of sodium/potassium sulfate and sodium pyrophosphate salts and the results were similar to that of MPBTA gels with mono-valent anions (Figure S17). pH values of the MPBTA and MTBTA gels were around 7.0, and we checked the gelation at different pH (4.0 to 9.0) by adding 1.0 to 3.0 N HCl or NaOH, respectively. Enhanced mechanical stability was observed for MPBTA gels in the presence of HCl (Figure S18), but partial gels were formed for MTBTA below 5.0 pH, and the mechanical strength of the MTBTA gels in the presence of HCl (pH = 5.0 to 6.0) was lower compared to the native gel (Figure S19). The experiments performed at basic conditions indicated that MPBTA formed gels with 1.0 N NaOH (pH = 8.0), and the mechanical strength was lower compared to neutral gels (Figure S18), but solutions were obtained at higher pH, and MTBTA did not form gels in basic conditions. We also tested the stability of MPBTA and MTBTA gels in the presence of halides and nitrates of calcium and magnesium salts, which indicated similar results, where the MPBTA gel remained intact in all cases but the MTBTA gel was disrupted. These results suggest that MPBTA showed a constructive interaction and MTBTA showed a destructive interaction towards anions. We have demonstrated the importance of choosing the functional groups where the anion-sensing properties can be switched from constructive to destructive interaction by simply altering the functional groups. This work illustrates the potential of developing stimuli-responsive supramolecular gels by modulating the non-covalent interaction.

## 3. Conclusions

The impact of additional hydrogen-bonding groups on the stimuli-responsive properties of supramolecular gels was analyzed by comparing the gelation properties of trimesic amides based on methyl esters of phenylalanine (MPBTA), and tyrosine (MTBTA) with additional hydroxy groups. The gelation studies indicated that MPBTA formed gels in alcohols and non-polar aromatic solvents, but the gelation experiments with MTBTA were unsuccessful. MTBTA was insoluble in apolar aromatic solvents, and a clear solution was obtained in other solvents. The gelation test in aqueous mixtures (1:1, *v*/*v*) of polar protic and aprotic solvents showed that MPBTA formed gels in an aqueous mixture of methanol, ethanol, DMF, and DMSO, whereas MTBTA formed gel only in DMF/water. The comparison of thermal and mechanical stability revealed that MPBTA gel was stronger than MTBTA gel. The morphology of the gel network was analyzed by SEM, which revealed that the chirality was transferred to gel fibers for MPBTA xerogel from *p*-xylene, *m*-xylene, and DMSO/water. The comparison of the MPBTA and MTBTA xerogels indicated that the additional hydroxy groups in MTBTA led to a minor morphological change. The PXRD patterns of bulk crystals and xerogels of MPBTA and MTBTA perfectly matched with the corresponding simulated patterns, indicating a similar mode of interactions in the crystal structure and the xerogels. Analysis of the stimuli-responsive properties of MPBTA and MTBTA towards sodium and potassium salts suggested that the gel network of MPBTA remained stable in the presence of halides, nitrate, sulfate, and pyrophosphate with enhanced mechanical and thermal stabilities compared to the native gelator, but the network was disrupted by cyanide salts. The experiments performed below MGC confirmed anion-induced gelation for MPBTA, but increasing the concentration of anions decreased the mechanical strength except in the presence of fluoride ions, indicating that the higher anion concentration disrupts the hydrogen-bonding interactions. However, MTBTA gels were disrupted by the presence of anions, which showed that MPBTA showed a constructive interaction and MTBTA showed a destructive interaction in the presence of anions. This work shows that the elegant choice of functional groups enables the design of stimuli-responsive materials with tunable properties.

## 4. Materials and Methods

The precursors, reagents, and solvents were obtained from commercial sources such as Sigma-Aldrich (MEDOR ehf, Reykjavik, Iceland), Fluorochem (Glossop, UK), and TCI-Europe (Boereveldseweg, Belgium) and were used in their received form. Deionized water was employed for the gelation tests. Characterization of the molecules was carried out using ^1^H and ^13^C NMR spectroscopy (Figures S20–S23), which were recorded on a Bruker Avance 400 spectrometer (Rheinstetten, Germany), and the SEM images (Carl Zeiss, Oberkochen, Germany) were captured on a Leo Supra 25 microscope. The rheological studies were conducted using an Anton Paar modular compact rheometer MCR 302 (Graz, Austria). Bruker D8 (Karlsruhe, Germany) and PANalytical (Almelo, Netherlands) X-ray diffraction (XRD) instruments were used in the single-crystal (SCXRD) and powder (PXRD) diffraction experiments, respectively. A JASCO J-1100 CD spectrometer (Tokyo, Japan) was used to conduct circular dichroism (CD) experiments and ATR–FTIR experiments were performed using a Nicolet iS50 FT-IR spectrometer (Gammadata Instrument AB, Uppsala, Sweden).

### 4.1. Synthesis of Ligands

#### 4.1.1. Synthesis of R,R,R-benzene-1,3,5-tricarboxamide of Phenylalanine Methyl Ester (MPBTA)

We synthesized MPBTA by modifying the reported procedure [42]. A 250 mL two-neck round-bottom flask was charged with *R*-phenylalanine methyl ester hydrochloride (2.44 g, 11.31 mmol) and 60.0 mL of dichloromethane under a nitrogen atmosphere. The mixture was cooled to 0 °C before adding triethylamine (4.72 mL, 33.90 mmol). After obtaining a clear solution, 1,3,5-benzenetricarbonyl trichloride (1.0 g, 3.77 mmol) in 30 mL dichloromethane was added dropwise to the reaction mixture and stirred overnight at room temperature. The solvent was removed through evaporation after completion of the reaction. The resulting crude mixture was left open under a fume hood for 24 h to remove triethylamine and subsequently treated with a 5.0% sodium bicarbonate solution for 12 h. The mixture was then filtered, thoroughly rinsed with water, and dried to yield the amide as a white powder. Finally, the product was recrystallized from ethanol. Yield: 1.87 g, 71.5%. ^1^H NMR (400 MHz, CDCl_3_) δ (ppm) 8.30 (s, 3H), 7.46–7.33 (m, 15H), 5.24–5.17 (m, 3H), 3.92 (s, 9H), 3.46–3.31 (m, 6H). ^13^C {^1^H} NMR (100 MHz, CDCl_3_) δ (ppm) 172.29, 165.21, 135.99, 134.66, 129.22, 128.72, 128.56, 127.26, 54.16, 52.60, 37.89. MS (ESI): calcd for C_39_H_39_N_3_O_9_Na [M + Na]^+^, 716.2579; found, 716.2567.

#### 4.1.2. Synthesis of R-tyrosine Methyl Ester Hydrochloride

The reported procedure was used to synthesize R-tyrosine methyl ester hydrochloride and the analytical data were in accordance with the reported data [60].

#### 4.1.3. Synthesis of R,R,R-benzene-1,3,5-tricarboxamide of Tyrosine Methyl Ester (MTBTA)

*R*-tyrosine methyl ester hydrochloride (2.2 g, 9.48 mmol) was dissolved in 50.0 mL of anhydrous dichloromethane by adding triethylamine (3.96 mL, 28.44 mmol) under a nitrogen atmosphere. Trimethylsilyl chloride (1.21 mL, 9.48 mmol) was then added, and the reaction mixture was diluted with an additional 50 mL of anhydrous dichloromethane to obtain a clear solution. After stirring the mixture at room temperature for 5 h, a solution of benzene-1,3,5-tricarbonyl trichloride (0.84 g, 3.16 mmol) in 30.0 mL of anhydrous dichloromethane was added dropwise at 0 °C and the resulting mixture was stirred overnight at room temperature. The solvent was evaporated using a rotary evaporator once the reaction was finished. The resulting crude mixture was left open under a fume hood for 24 h to eliminate triethylamine and subsequently treated with a 5.0% sodium bicarbonate solution for 12 h. After filtering, rinsing with water, and drying, the amide was obtained as a white powder. The product was then recrystallized using a mixture of ethanol/water (2:1 *v*/*v*). Yield: 1.82 g, 75.8%. ^1^H NMR (400 MHz, DMSO-d6) δ (ppm): 9.21 (s, 3H), 9.12 (d, J = 7.6 Hz, 3H), 8.38 (s, 3H), 7.10–7.06 (m, 6H), 6.68–6.64 (m, 6H), 4.60 (ddd, J = 9.2, 7.6, 6.0 Hz, 3H), 3.62 (s, 9H), 3.03 (h, J = 5.4 Hz, 6H). ^13^C {^1^H} NMR (100 MHz, DMSO-d6) δ (ppm): 172.62, 166.10, 156.43, 134.71, 130.44, 129.79, 128.03, 115.59, 55.41, 52.40, 36.00. MS (ESI): calcd for C_39_H_39_N_3_O_12_Na [M + Na]^+^, 764.2426; found, 764.2417.

### 4.2. Circular Dichroism (CD)

A JASCO J-1100 CD spectrometer (Tokyo, Japan) was used to collect the data in a continuous scanning mode with a wavelength range of 200 to 400 nm at a 50.0 nm/minute rate and 1.0 nm bandwidth. The solution-state CD experiments on MPBTA and MTBTA were carried out in ethanol with the enantiomeric and equimolar chiral compounds at different concentrations (0.01, 0.015, 0.02, and 0.03 wt/v%). The best results were obtained at 0.015 wt/v %, which was taken as the optimum concentration, where the HT value was observed to be optimal. For MPBTA, positive peaks were observed at around 205 nm and 235 nm, which was compared with the enantiomer of MPBTA, where a mirror image of the spectrum was observed (Figure S24). Similarly, MTBTA showed positive peaks at 205 nm and 240 nm, and the enantiomer of MTBTA showed a mirror image (Figure S24), indicating the preservation of chirality.

### 4.3. Gelation Studies

To make the gelation mixture, a standard 7.0 mL vial with a 15.0 mm inner diameter was charged with 10.0 mg of the gelator and the corresponding solvent (1.0 mL). The vial was then tightly closed. The mixture underwent sonication (30–60 s) and gradual heating until a transparent solution formed, which was carried out by heating (~3.0 min) with a heat gun. This solution was allowed to age for 24 h, and gelation was verified by inverting the vial. Gelation experiments in a 1:1 *v*/*v* mixed aqueous system were conducted by dissolving the 10.0 mg of gelator in 0.5 mL of solvent and adding 0.5 mL of deionized water. We then followed the same sonication and gradual heating procedure to form a clear solution, which was left untouched to gelate, and the gel formation was verified by turning the vial upside down after 24 h. If a partial gel or clear solution was observed, we repeated the experiments at a higher quantity of gelator, increasing up to 70.0 mg, to ensure gel formation.

#### 4.3.1. Minimum Gelator Concentration (MGC)

To determine the minimum gelator concentration (MGC) of a gelator, we charged standard 7.0 mL vials with different quantities of a gelator, ranging from lower to higher quantities, and followed the above procedure for gel formation. After 24 h, the MGC was determined to be the lowest necessary quantity of the gelator to establish a stable gel network.

#### 4.3.2. *T_gel_* Experiments

A standard 7.0 mL vial was filled with an adequate amount of gelator and 1.0 mL of solvent. The mixture was heated and sonicated to make a clear solution and left to age for one day. A small, spherical glass ball (100.0 mg) was cautiously positioned on the gel surface. The vial was tightly closed and submerged in an oil bath equipped with a magnetic stirrer and a temperature sensor. The temperature of the oil bath was increased gradually at a rate of approximately 10.0 °C per minute. The temperature at which the glass ball sank to the bottom of the vial was recorded as *T_gel_*. Three trials were executed for each concentration, and the average value was calculated.

### 4.4. Rheology

Two different methods were used to prepare the gel for rheological studies: vial and syringe. MPBTA gel in *p*-xylene (6.5 wt/v%) was prepared in a vial. A mixture containing the gelator at 6.5 wt/v% and 1.0 mL of *p*-xylene was heated and sonicated until a clear solution was obtained. After 24 h, the resulting gel was scooped out onto the rheometer using a spoon, and rheology was performed on the gel. For the syringe method, the gels were prepared in DMF/water (1:1, *v*/*v*) and DMSO/water (1:1, *v*/*v*) at 6.5 wt/v%. The tip of the syringe was cut off and the hot solution containing the gelator at 6.5 wt/v% was poured into the syringe body and left to gel. The resulting gel was pressed out of the syringe onto the rheometer after 24 h. The rheology of the gel was then measured. An Anton Paar Modular Compact Rheometer (MCR) was used for rheological studies with a 1.0 mm gap height. During the frequency and amplitude sweeps, a Peltier temperature control hood maintained a constant temperature of 20.0 °C by acting as a solvent trap. The amplitude sweep was conducted with a constant frequency of 1.0 Hz and a logarithmic ramp strain (Y) ranging from 0.01 to 100%. The frequency sweep was carried out within the 0.1 to 10.0 Hz range, specifically within the linear viscoelasticity domain (0.01% strain). For each sample, the measurements were taken three times.

### 4.5. Scanning Electron Microscopy (SEM)

We prepared MPBTA gels in *p*-xylene (1.0 wt/v%), *m*-xylene (3.0 wt/v%), DMF/water (1:1, *v*/*v*, at 6.5 wt/v%), and DMSO/water (1:1, *v*/*v*, at 3.0 wt/v%), and MTBTA in DMF/water (1:1, *v*/*v*, at 6.5 wt/v%). The gels were filtered after 24 h and air-dried to obtain the xerogels. The surface morphologies of the xerogels were explored using scanning electron microscopy (SEM) images captured using a Leo Supra 25 microscope. A pin mount was used to hold a small amount of xerogel, and the carbon tab was affixed to the top. In order to prevent charging, a layer of gold (12.0 nm thickness) was applied to the surface and maintained for 5–6 min before loading. The images were captured with an operating voltage of 3.0 kV and a working distance ranging from 3.0 to 4.0 mm. The SEM images were captured using an in-lens detector.

### 4.6. Single-Crystal X-ray Diffraction (SCXRD)

The X-ray diffraction analysis was performed using a Bruker D8 Venture diffractometer with a Photon100 CMOS detector and open-flow nitrogen cryostats from Cryostream (Oxford Cryosystems, Long Hanborough, Oxford, UK). Prior to mounting, X-ray-quality crystals were isolated and immersed in paratone oil. The determination of the unit cell, collecting data, data reduction, absorption correction, and structure solution/final refinements were carried out in apex-III software APEX3V2016.9-0 (Bruker AXS: Madison, WI, 2015). The structure was solved using the direct methods, and SHELXTL’s full-matrix least squares on F^2^ were used to refine the data. The disordered atoms of the methoxy groups were refined using a free variable (FVAR), and anisotropic refinement was performed on all atoms except for the hydrogen atoms. The hydrogen atoms were placed in their calculated position, and a riding model was used to refine these atoms. The crystallographic data are deposited at the Cambridge Crystallographic Data Centre (CCDC number: 2374952) and can be obtained free of charge.

### 4.7. Powder X-ray Diffraction

We conducted PXRD analysis on the bulk crystals of MPBTA and MTBTA, which were obtained from ethanol and ethanol/water mixture (2:1, *v*/*v*), respectively. The xerogels of MPBTA were prepared by drying the gels from an aqueous mixture of DMF and DMSO (1:1, *v*/*v*) at a concentration of 5.0 wt/v% and from *p*-xylene at the same concentration. Similarly, the xerogel of MTBTA was prepared from a DMF/water mixture (1:1, *v*/*v*) at a concentration of 5.0 wt/v%. The resulting gel was then scooped out onto a piece of filter paper and left to air-dry. The resulting xerogel was then ground into a powder, and a PXRD was conducted.

### 4.8. Infrared Spectroscopy

ATR–FTIR was measured using a Nicolet iS50 FT-IR spectrometer (Tokyo, Japan). The crystals, xerogels of MPBTA (2.0 wt/v%) and MTBTA (4.5 wt/v%) from DMF/water (1:1, *v*/*v*), and gels of MPBTA (2.0 wt/v%) and MTBTA (4.5 wt/v%) in DMF/D_2_O (1:1, *v*/*v*) were used. Samples were directly placed onto the ATR crystal and spectra were recorded over a range of 3400–400 cm^−1^.

### 4.9. Stimuli-Responsive Properties

A total of 14.0 mg of MPBTA (at MGC) was dissolved in 0.5 mL of DMF. The aqueous solutions (0.5 mL) of sodium/potassium halides, nitrate, sulfate, pyrophosphate, and cyanide (1.0 equiv.) were added and heated to produce a clear solution, and the solution then remained undisturbed to facilitate gel formation. The thermal properties of the gels were analyzed using *T_gel_* studies, and the mechanical properties were recorded using rheological measurements (frequency sweep) in accordance with the procedures (syringe method) outlined in Section 4.3.2 and Section 4.4, respectively. To test the effect of anions at a concentration below MGC, gels of MPBTA were prepared at 1.0 wt/v% with sodium/potassium salts (1.0 equiv.) We also performed experiments above MGC (1.8 wt/v%) using 1.0 and 3.0 equiv. of sodium/potassium salts. Rheology and *T_gel_* studies were conducted on these gels. MTBTA gels were prepared by mixing 45.0 mg (at MGC) in 0.5 mL DMF and 1.0 equivalents of sodium/potassium salts. The experiments were performed below MGC using 40.0 mg of the MTBTA. For each anion, the experiments were conducted twice.

## Figures and Tables

**Table 1:** MGC (wt/v%) and *T_gel_* (°C) values of MPBTA and MTBTA at 6.5 wt/v%.

Solvent	MPBTA		MTBTA	
	MGC	*T_gel_*	MGC	*T_gel_*
DMF/H_2_O (1:1, *v*/*v*)	1.4	93.3	4.5	69.2
DMSO/H_2_O (1:1, *v*/*v*)	0.7	119.4	Crystals *	-
*p*-xylene	1.0	121.6	Insoluble	-
ethanol	4.0	67.8	Solution **	-

* 8.0 wt/v% and ** 6.0 wt/v%.

## Data Availability

All the details are given in the Supplementary Materials.

## Supplementary Materials

The following supporting information can be downloaded at: https://www.mdpi.com/article/10.3390/gels10090584/s1, Figure S1: Amplitude-sweep measurement performed on the MPBTA and MTBTA gels in DMF/water (1:1, *v*/*v*) at 6.5 wt/v% using syringe method; Figure S2: Comparison of mechanical strength of MPBTA gels at 6.5 wt/v% in 1:1 (*v*/*v*) mixture of DMF/water and DMSO/water obtained from scooping and syringe method; Figure S3: Photo showing (a) MPBTA gel in *p*-xylene coating the walls of a syringe and (b) dried MPBTA gel in *p*-xylene; Figure S4: SEM images of MPBTA xerogels from *m*-xylene at 3.0 wt/v% (thickness 0.2–3.2 µm); Figure S5: Comparison of PXRD patterns of MPBTA bulk crystals and simulated pattern of single crystal; Figure S6: Comparison of PXRD patterns of MPBTA xerogels from *p*-xylene, DMF/water (1:1, *v*/*v*), and DMSO/water (1:1, *v*/*v*) at 5.0 wt/v% and simulated pattern of single crystal; Figure S7: IR spectra of MPBTA crystals (top) and xerogel (bottom) from DMF/H_2_O (1:1, *v*/*v*) at 2.0 wt/v%; Figure S8: IR spectra of MPBTA gel (bottom) in DMF/D_2_O (1:1, *v/v*) at 2.0 wt/v % and DMF/D_2_O (1:1, *v*/*v*) mixture (top); Figure S9: IR spectra of MTBTA: crystal (top) and xerogel (bottom) from DMF/H_2_O (1:1, *v*/v) at 4.5 wt/v %; Figure S10: IR spectra of MTBTA gel (bottom) in DMF/D_2_O (1:1, *v*/*v*) at 4.5 wt/v % and DMF/D_2_O (1:1, *v/v*) mixture (top); Figure S11: Stimuli-responsive properties of the MPBTA gels at MGC (1.4 wt/v%) in DMF/water mixture (1:1, *v*/*v*) with potassium salts; Figure S12: Frequency-sweep experiments of MPBTA gels at MGC (1.4 wt/v%) in the presence of various sodium/potassium salts (1.0 equiv.) of halides and nitrate; Figure S13: Frequency-sweep experiments of MPBTA gels above MGC (1.8 wt/v%) in the presence of various sodium salts (1.0 equiv. and 3.0 equiv.) of halides and nitrate; Figure S14: Frequency-sweep experiments of MPBTA gels above MGC (1.8 wt/v%) in the presence of various potassium salts (1.0 equiv. and 3.0 equiv.) of halides and nitrate; Figure S15: Stimuli-responsive properties of the MTBTA gels in DMF/water mixture (1:1, *v/v*) with potassium salts (1.0 equiv.); Figure S16: Stimuli-responsive properties of (a) MPBTA gels (1.4 wt/v%) and (b) MTBTA gels (4.5 wt/v%) in DMF/water mixture (1:1, *v*/*v*) towards multi-valent anions of sodium and potassium salts; Figure S17: Frequency-sweep experiments of MPBTA gels above MGC (1.8 wt/v%) in the presence of multi-valent anions of sodium and potassium salts (1.0 equiv. and 3.0 equiv.); Figure S18: Frequency-sweep experiments of MPBTA gels at MGC (1.4 wt/v%) prepared at different pH; Figure S19: Frequency-sweep experiments of MTBTA gels at MGC (4.5 wt/v%) prepared at different pH; Figure S20: ^1^H NMR spectrum of MPBTA; Figure S21: ^13^C NMR spectrum of MPBTA; Figure S22: ^1^H NMR spectrum of MTBTA; Figure S23: ^13^C NMR spectrum of MTBTA; Figure S24: CD spectra for MPBTA and MTBTA compounds (top) and the corresponding HT data (bottom) in the solution state at 0.015 wt/v% in absolute EtOH; Scheme S1: Synthesis of MPBTA and MTBTA; Table S1: Gelation Experiments; Table S2: Determination of Minimum Gelator Concentration (MGC); Table S3: Crystal data of MTBTA; Table S4: Comparison of IR spectra of MPBTA and MTBTA (cm^−1^); Table S5: *T_gel_* studies with MPBTA gels at MGC (1.4 wt/v%) in DMF/water (1:1, *v/v*), in the presence of 1.0 equivalents of sodium and potassium salts; Table S6: *T_gel_* studies with MPBTA gels at MGC (1.4 wt/v%) in DMF/water (1:1, *v*/*v*), in the presence of 1.0 equivalents of multi-valent anions of sodium and potassium salts.

## References

[1] de Loos M., Feringa B.L., van Esch J.H. (2005). Design and Application of Self-Assembled Low Molecular Weight Hydrogels. Eur. J. Org. Chem..

[2] Kumar D.K., Steed J.W. (2014). Supramolecular gel phase crystallization: Orthogonal self-assembly under non-equilibrium conditions. Chem. Soc. Rev..

[3] Smith D.K. (2024). Supramolecular gels—A panorama of low-molecular-weight gelators from ancient origins to next-generation technologies. Soft Matter.

[4] Adams D.J. (2022). Personal Perspective on Understanding Low Molecular Weight Gels. J. Am. Chem. Soc..

[5] Jones C.D., Steed J.W. (2016). Gels with sense: Supramolecular materials that respond to heat, light and sound. Chem. Soc. Rev..

[6] Li L., Sun R., Zheng R., Huang Y. (2021). Anions-responsive supramolecular gels: A review. Mater. Des..

[7] Panja S., Adams D.J. (2021). Stimuli responsive dynamic transformations in supramolecular gels. Chem. Soc. Rev..

[8] Chu C.-W., Schalley C.A. (2021). Recent Advances on Supramolecular Gels: From Stimuli-Responsive Gels to Co-Assembled and Self-Sorted Systems. Org. Mater..

[9] Yang X., Zhang G., Zhang D. (2012). Stimuli responsive gels based on low molecular weight gelators. J. Mater. Chem..

[10] Lloyd G.O., Steed J.W. (2009). Anion-tuning of supramolecular gel properties. Nat. Chem..

[11] Yu G., Yan X., Han C., Huang F. (2013). Characterization of supramolecular gels. Chem. Soc. Rev..

[12] Draper E.R., Adams D.J. (2017). Low-Molecular-Weight Gels: The State of the Art. Chem.

[13] Terech P., Weiss R.G. (2006). Molecular Gels: Materials with Self-Assembled Fibrillar Networks.

[14] Fages F., Vögtle F., Žinic M. (2005). Systematic Design of Amide- and Urea-Type Gelators with Tailored Properties. Low Molecular Mass Gelator.

[15] Moulin E., Armao J.J., Giuseppone N. (2019). Triarylamine-Based Supramolecular Polymers: Structures, Dynamics, and Functions. Acc. Chem. Res..

[16] Wang Y., de Kruijff R.M., Lovrak M., Guo X., Eelkema R., van Esch J.H. (2019). Access to Metastable Gel States Using Seeded Self-Assembly of Low-Molecular-Weight Gelators. Angew. Chem. Int. Ed..

[17] Ghosh D., Farahani A.D., Martin A.D., Thordarson P., Damodaran K.K. (2020). Unraveling the Self-Assembly Modes in Multicomponent Supramolecular Gels Using Single-Crystal X-ray Diffraction. Chem. Mater..

[18] Kuppadakkath G., Jayabhavan S.S., Damodaran K.K. (2024). Supramolecular Gels Based on C3-Symmetric Amides: Application in Anion-Sensing and Removal of Dyes from Water. Molecules.

[19] Dastidar P. (2008). Supramolecular gelling agents: Can they be designed?. Chem. Soc. Rev..

[20] Estroff L.A., Hamilton A.D. (2004). Water Gelation by Small Organic Molecules. Chem. Rev..

[21] Ghosh D., Chaudhary P., Pradeep A., Singh S., Rangasamy J., Damodaran K.K. (2023). Structural modification induced hydrogelation and antibacterial properties in supramolecular gels. J. Mol. Liq..

[22] Jayabhavan S.S., Kristinsson B., Ghosh D., Breton C., Damodaran K.K. (2023). Stimuli-Responsive Properties of Supramolecular Gels Based on Pyridyl-N-oxide Amides. Gels.

[23] Sudhakaran Jayabhavan S., Ghosh D., Damodaran K.K. (2021). Making and Breaking of Gels: Stimuli-Responsive Properties of Bis(Pyridyl-N-oxide Urea) Gelators. Molecules.

[24] Martínez-Máñez R., Sancenón F. (2003). Fluorogenic and Chromogenic Chemosensors and Reagents for Anions. Chem. Rev..

[25] Bowman-James K. (2005). Alfred Werner Revisited:  The Coordination Chemistry of Anions. Acc. Chem. Res..

[26] Gunnlaugsson T., Glynn M., Tocci G.M., Kruger P.E., Pfeffer F.M. (2006). Anion recognition and sensing in organic and aqueous media using luminescent and colorimetric sensors. Coord. Chem. Rev..

[27] Gale P.A., García-Garrido S.E., Garric J. (2008). Anion receptors based on organic frameworks: Highlights from 2005 and 2006. Chem. Soc. Rev..

[28] Jose D.A., Kumar D.K., Ganguly B., Das A. (2007). Rugby-Ball-Shaped Sulfate–Water–Sulfate Adduct Encapsulated in a Neutral Molecular Receptor Capsule. Inorg. Chem..

[29] Beer P.D., Gale P.A. (2001). Anion Recognition and Sensing: The State of the Art and Future Perspectives. Angew. Chem. Int. Ed..

[30] Rahmati N., Hoebeek F.E., Peter S., De Zeeuw C.I. (2018). Chloride Homeostasis in Neurons With Special Emphasis on the Olivocerebellar System: Differential Roles for Transporters and Channels. Front. Cell. Neurosci..

[31] Şan, Dey A.K., Giri B. (2016). Fluoride Fact on Human Health and Health Problems: A Review. Med. Clin. Rev..

[32] Hendry-Hofer T.B., Ng P.C., Witeof A.E., Mahon S.B., Brenner M., Boss G.R., Bebarta V.S. (2019). A Review on Ingested Cyanide: Risks, Clinical Presentation, Diagnostics, and Treatment Challenges. J. Med. Toxicol..

[33] Moss B. (2008). Water pollution by agriculture. Philos. Trans. R. Soc. Lond. Ser. B Biol. Sci..

[34] Evans N.H., Beer P.D. (2014). Advances in Anion Supramolecular Chemistry: From Recognition to Chemical Applications. Angew. Chem. Int. Ed..

[35] Chang S.G., Littlejohn D., Hu K.Y. (1987). Disulfate Ion as an Intermediate to Sulfuric Acid in Acid Rain Formation. Science.

[36] Busschaert N., Caltagirone C., Van Rossom W., Gale P.A. (2015). Applications of Supramolecular Anion Recognition. Chem. Rev..

[37] Panja A., Ghosh K. (2018). Pyridylazo Derivatives with Dicyanovinyl Appendage in Selective Sensing of CN^−^ in Sol-Gel Medium. ChemistrySelect.

[38] Desiraju G.R. (1995). Supramolecular Synthons in Crystal Engineering—A New Organic Synthesis. Angew. Chem. Int. Ed. Engl..

[39] Sudhakaran Jayabhavan S., Kuppadakkath G., Damodaran K.K. (2023). The Role of Functional Groups in Tuning the Self-Assembly Modes and Physical Properties of Multicomponent Gels. ChemPlusChem.

[40] Bondy C.R., Loeb S.J. (2003). Amide based receptors for anions. Coord. Chem. Rev..

[41] Molina P., Zapata F., Caballero A. (2017). Anion Recognition Strategies Based on Combined Noncovalent Interactions. Chem. Rev..

[42] Ishioka Y., Minakuchi N., Mizuhata M., Maruyama T. (2014). Supramolecular gelators based on benzenetricarboxamides for ionic liquids. Soft Matter.

[43] Panja S., Panja A., Ghosh K. (2021). Supramolecular gels in cyanide sensing: A review. Mater. Chem. Front..

[44] Aletti A.B., Blasco S., Aramballi S.J., Kruger P.E., Gunnlaugsson T. (2019). Sulfate-Templated 2D Anion-Layered Supramolecular Self-Assemblies. Chem.

[45] Ghosh A., Das P., Kaushik R., Damodaran K.K., Jose D.A. (2016). Anion responsive and morphology tunable tripodal gelators. RSC Adv..

[46] Malviya N., Das M., Mandal P., Mukhopadhyay S. (2017). A smart organic gel template as metal cation and inorganic anion sensor. Soft Matter.

[47] Liu J., Yang H.-L., Sun X.-W., Zhang Y.-M., Yao H., Wei T.-B., Lin Q. (2021). A simple pillar[5]arene assembled multi-functional material with ultrasensitive sensing, self-healing, conductivity and host–guest stimuli-responsive properties. Soft Matter.

[48] Zhao Q., Dai X.-Y., Yao H., Zhang Y.-M., Qu W.-J., Lin Q., Wei T.-B. (2021). Stimuli-responsive supramolecular hydrogel with white AIE effect for ultrasensitive detection of Fe3+ and as rewritable fluorescent materials. Dye. Pigments.

[49] de Windt L.N.J., Fernández Z., Fernández-Míguez M., Freire F., Palmans A.R.A. (2022). Elucidating the Supramolecular Copolymerization of N- and C-Centered Benzene-1,3,5-Tricarboxamides: The Role of Parallel and Antiparallel Packing of Amide Groups in the Copolymer Microstructure. Chem. Eur. J..

[50] Gudmundsson T.A., Kuppadakkath G., Ghosh D., Ruether M., Seddon A., Ginesi R.E., Doutch J., Adams D.J., Gunnlaugsson T., Damodaran K.K. (2024). Nanoscale assembly of enantiomeric supramolecular gels driven by the nature of solvents. Nanoscale.

[51] Prasad K. (2019). Rheology for Chemists—An Introduction. Appl. Rheol..

[52] Guenet J.-M. (2016). Organogels: Thermodynamics, Structure, Solvent Role, and Properties.

[53] Adams D.J. (2018). Does Drying Affect Gel Networks?. Gels.

[54] Srinivasulu G., Sridhar B., Ravi Kumar K., Sreedhar B., Ramesh V., Srinivas R., Kunwar A.C. (2011). Molecular self assembly of benzene-1,3,5-tricarbonyl phenylalanine. J. Mol. Struct..

[55] Jana P., Paikar A., Bera S., Maity S.K., Haldar D. (2014). Porous Organic Material from Discotic Tricarboxyamide: Side Chain–Core interactions. Org. Lett..

[56] Fornaro T., Burini D., Biczysko M., Barone V. (2015). Hydrogen-Bonding Effects on Infrared Spectra from Anharmonic Computations: Uracil–Water Complexes and Uracil Dimers. J. Phys. Chem. A.

[57] Abul-Haija Y.M., Roy S., Frederix P.W.J.M., Javid N., Jayawarna V., Ulijn R.V. (2014). Biocatalytically Triggered Co-Assembly of Two-Component Core/Shell Nanofibers. Small.

[58] Chevigny R., Sitsanidis E.D., Schirmer J., Hulkko E., Myllyperkiö P., Nissinen M., Pettersson M. (2023). Nanoscale Probing of the Supramolecular Assembly in a Two-Component Gel by Near-Field Infrared Spectroscopy. Chem. Eur. J..

[59] Nebot V.J., Armengol J., Smets J., Prieto S.F., Escuder B., Miravet J.F. (2012). Molecular Hydrogels from Bolaform Amino Acid Derivatives: A Structure–Properties Study Based on the Thermodynamics of Gel Solubilization. Chem. Eur. J..

[60] Morishita Y., Kaino T., Okamoto R., Izumi M., Kajihara Y. (2015). Synthesis of D,L-amino acid derivatives bearing a thiol at the β-position and their enzymatic optical resolution. Tetrahedron Lett..

