# Supplementary material for: Designing Stimuli-Responsive Supramolecular Gels by Tuning the Non-Covalent Interactions of the Functional Groups
Source: Gels. 2024 Sep 11;10(9):584. doi: 10.3390/gels10090584 (PMC11430835; doi:10.3390/gels10090584)
Supplement: Supplementary file 1 [file gels-10-00584-s001.zip › Supplementary Materials/Gels_BTA_Gels2024_Supportinginfo_revised-1.pdf]

SUPPORTING INFORMATION

Designing Stimuli-responsive Supramolecular Gels by Tuning the Non-covalent Interactions of the Functional Groups

Geethanjali Kuppadakkath, Ira Volkova, and Krishna K. Damodaran*

Contents

1. Scheme for the synthesis……………………………………………………………………………… 2
2. Gelation studies…………………………………………………………………………………………… 3
3. Rheology……………………………………………………………………………………………………… 4
4. Scanning electron microscopy………………………………………………………………………… 5
5. Single crystal X-ray diffraction………………………………………………………………………… 6
6. Powder X-ray diffraction………………………………………………………………………………… 7
7. Infra-red Spectroscopy…………………………………………………………………………………… 8
8. Stimuli responsive properties………………………………………………………………………… 12
9. NMR spectra………………………………………………………………………………………………… 18
10. Circular dichroism………………………………………………………………………………………… 20

1. Synthesis of MPBTA and MTBTA compounds

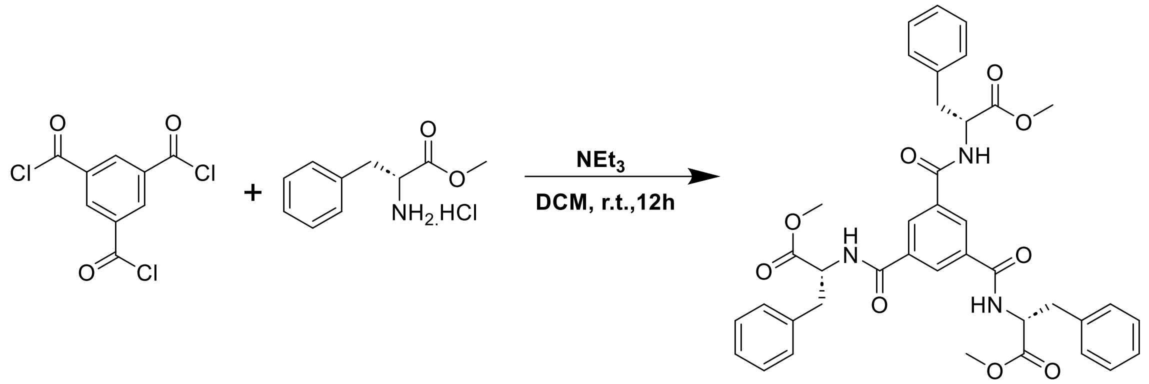

MPBTA

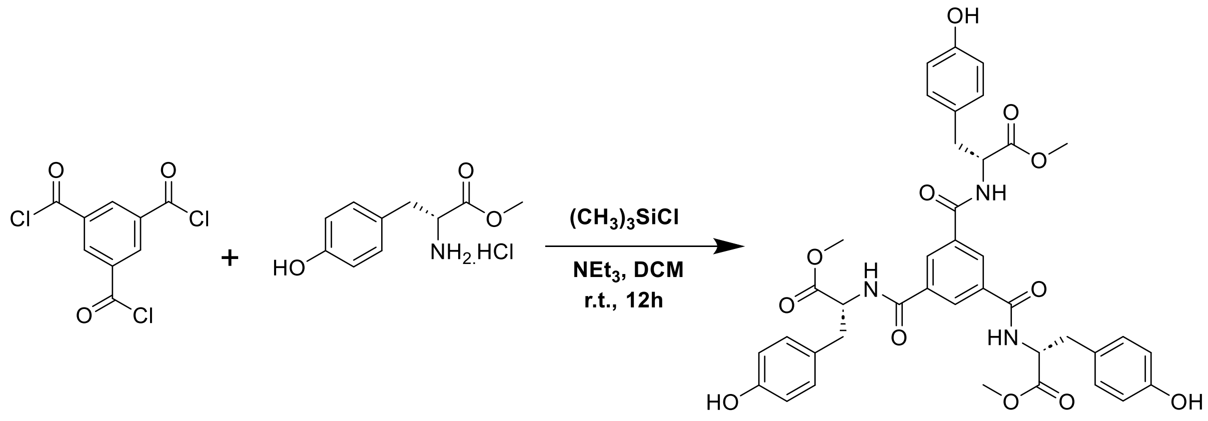

MTBTA

Scheme S1. Synthesis of MPBTA [1] and MTBTA.

[1] Ishioka, Y.; Minakuchi, N.; Mizuhata, M.; Maruyama, T. Supramolecular gelators based on benzenetricarboxamides for ionic liquids. *Soft Matter* **2014**, *10*, 965-971.

2. Gelation studies

Table S1. Gelation Experiments

Solvent	Gelation test at 1.0 wt/v%	
	MPBTA	MTBTA
Methanol	$G^{\$}$	S
Ethanol	G^{**}	S
Isopropanol	G^{*}	S
n-propanol	G^{*}	S
n-butanol	G^{*}	S
2-butanol	G^{*}	S
n-pentanol	G^{*}	P
p-xylene	G	I
m-xylene	G^{**}	I
o-xylene	G^{*}	I
Mesitylene	$G^{\#}$	I
Toluene	S	I
acetonitrile	S	S
dichloromethane	S	I
THF	S	S
ethyl acetate	S	I
chloroform	S	I
acetone	S	S
DMSO/H_2O (1:1, v/v)	G	PG
DMF/H_2O (1:1, v/v)	G^{*}	$G^{\#}$
EtOH/H_2O (1:1, v/v)	G	C
MeOH/H_2O (2:1, v/v)	G	C
THF/H_2O (1:1, v/v)	Sph	C

G= gel, I= insoluble, G^{*}= 2.0 wt/v%, G^{**}= 4.0 wt/v%, $G^{\#}$= 5.0 wt/v%.$G^{\$}$= 7.0 wt/v%, C= crystals, PG= partial gel, Sph= spherulites, S = solution and P = precipitate.

Table S2. Determination of Minimum Gelator Concentration (MGC)

Solvent	MGC (wt/v%)	
	**MPBTA**	**MTBTA**
Methanol	6.5	Solution*
Isopropanol	1.4	Solution*
n-propanol	2.0	Solution*
2-butanol	1.0	Solution*
m-xylene	2.2	Insoluble
Mesitylene	4.3	Insoluble
EtOH/water	0.7	Crystals*

* 6.0 wt/v%

3. Rheology

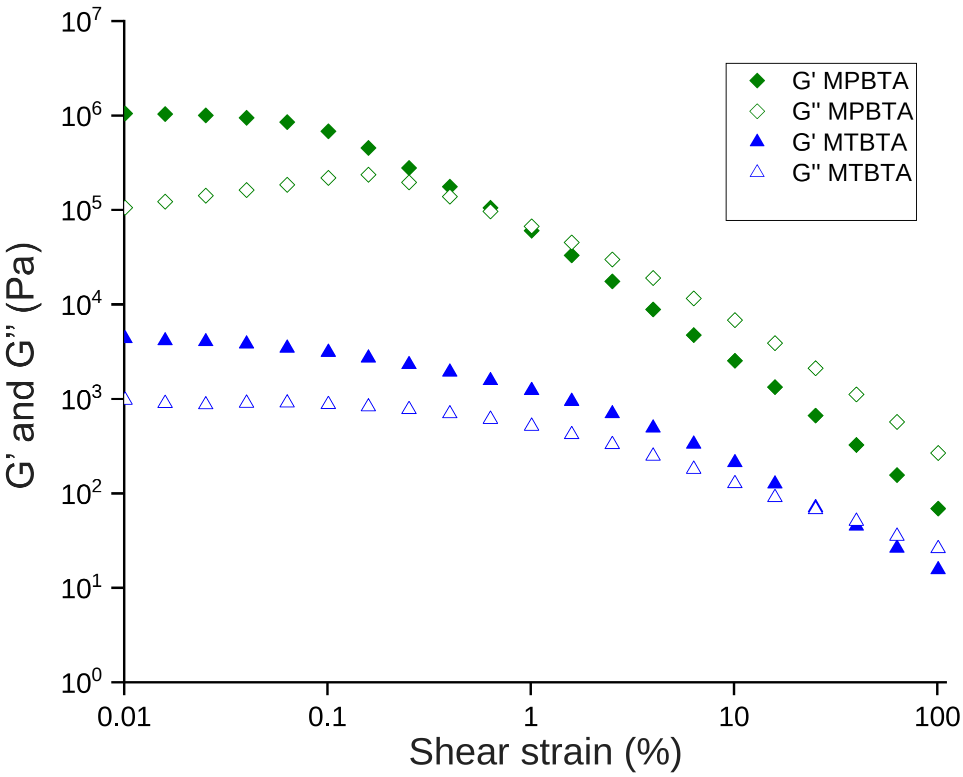

Figure S1. Amplitude sweep measurement performed on the MPBTA and MTBTA gels in DMF/water (1:1, v/v) at 6.5 wt/v% using syringe method.

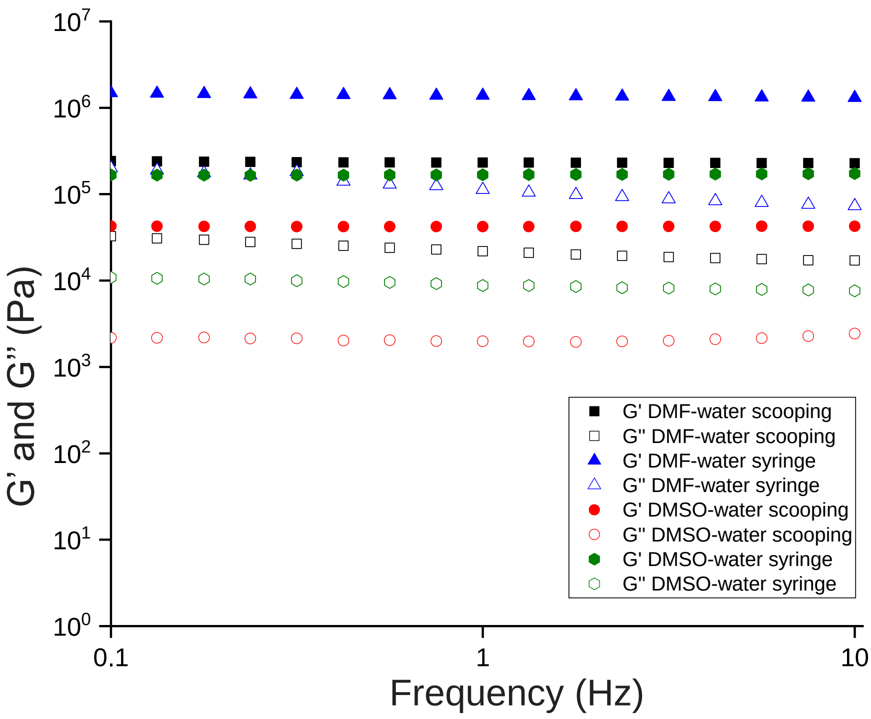

Figure S2. Comparison of mechanical strength of MPBTA gels at 6.5 wt/v% in 1:1 (v/v) mixture of DMF/water and DMSO/water obtained from scooping and syringe method.

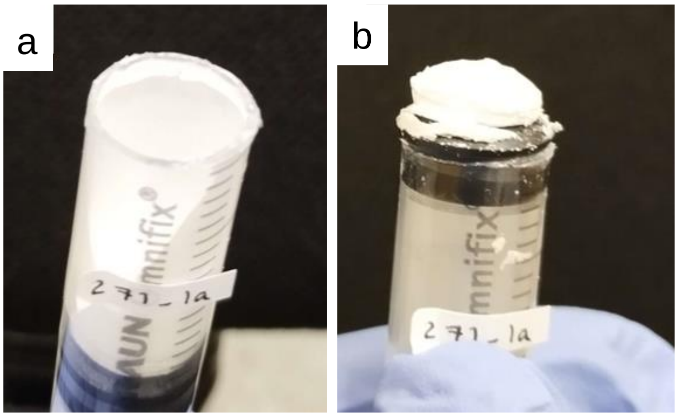

Figure S3. Photo showing (a) MPBTA gel in *p*-xylene coating the walls of a syringe and (b) dried MPBTA gel in *p*-xylene.

4. Scanning Electron Microscopy (SEM)

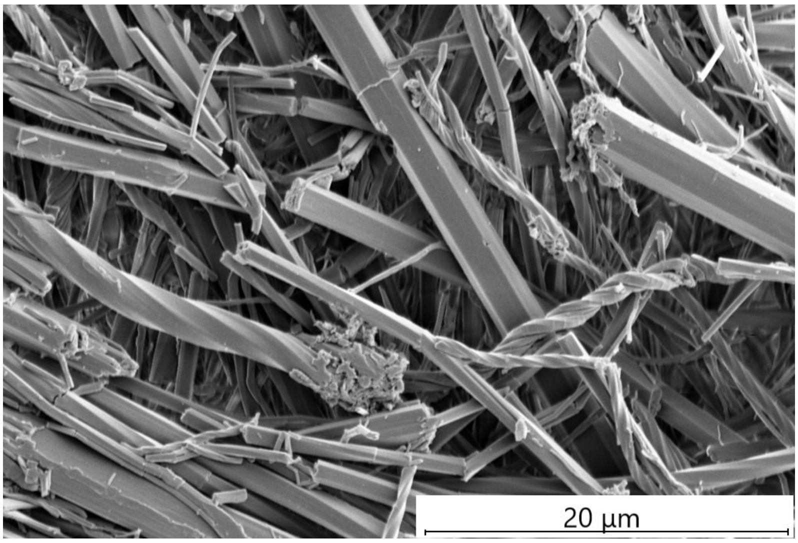

Figure S4. SEM images of MPBTA xerogels from *m*-xylene at 3.0 wt/v% (thickness 0.2- 3.2 µm).

5. Single crystal X-ray diffraction

Table S3: Crystal data of MTBTA

Crystal data	MTBTA
Empirical formula	$C_{39}H_{39}N_3O_{12}$
Color	Colorless
Formula weight	741.73
Crystal size (mm)	0.28 x 0.08 x 0.04
Crystal system	Monoclinic
Space group	$P2_1$
a (Å)	9.1493(8)
b (Å)	16.3156(13)
c (Å)	13.1041(11)
α (°)	90
β (°)	106.680(3)
γ (°)	90
Volume (Å^3)	1873.8(3)
Z	2
$D_{calc.}$(g/cm^3)	1.315
F(000)	780
μ (mm^{-1}) $MoK_α$	0.098
Temperature (K)	298(2)
Reflections collected/ unique/observed [I>2σ(I)]	43608/ 7428/5037
Data/restraints/parameters	7428/1/511
Goodness of fit on F^2	1.041
Final R indices [I>2σ(I)]	R_1 = 0.0487<br>wR_2 = 0.0883
R indices (all data)	R_1 = 0.0995<br>wR_2 = 0.1041

6. Powder X-ray diffraction

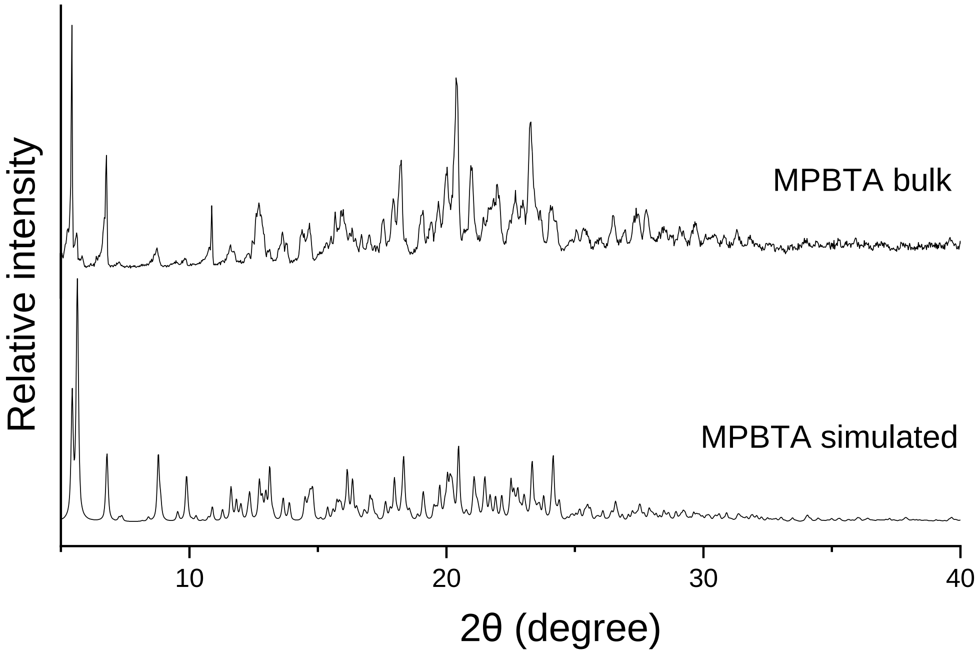

Figure S5. Comparison of PXRD patterns of MPBTA bulk crystals and simulated pattern of single crystal.

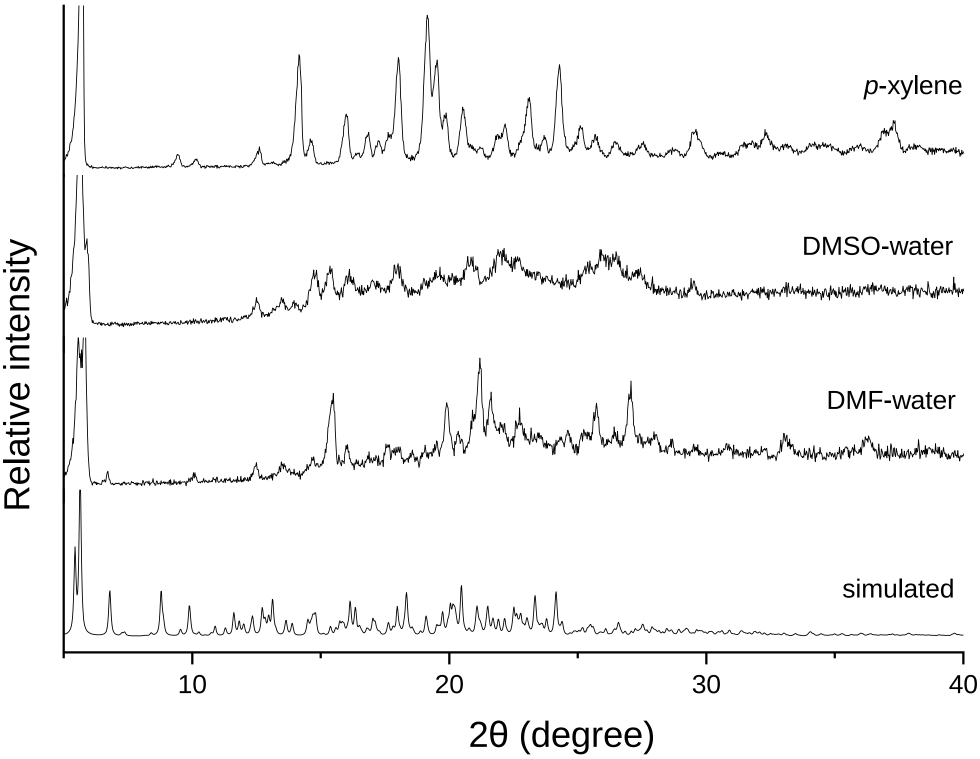

Figure S6. Comparison of PXRD patterns of MPBTA xerogels from *p*-xylene, DMF/water (1:1, v/v), and DMSO/water (1:1, v/v) at 5.0 wt/v% and simulated pattern of single crystal.

7. Infra-red Spectroscopy

Table S4: Comparison of IR spectra of MPBTA and MTBTA (cm^{-1})

Bond	MPBTA crystal	MPBTA gel	MPBTA xerogel	MTBTA crystal	MTBTA gel	MTBTA xerogel
Phenol (O-H Stretching)	-	-	-	3368	3064	3331
Amide (N-H stretching)	3285, 3402	3063, 3028	3225	3085	2936	3228
Ester (C=O stretching)	1736	1745	1744	1718, 1755	1720, 1742	1720, 1741
Amide (C=O stretching)	1638	1637	1636	1652	1648	1635
Amide (N-H bending)	1530	1459	1539	1613	1513	1547

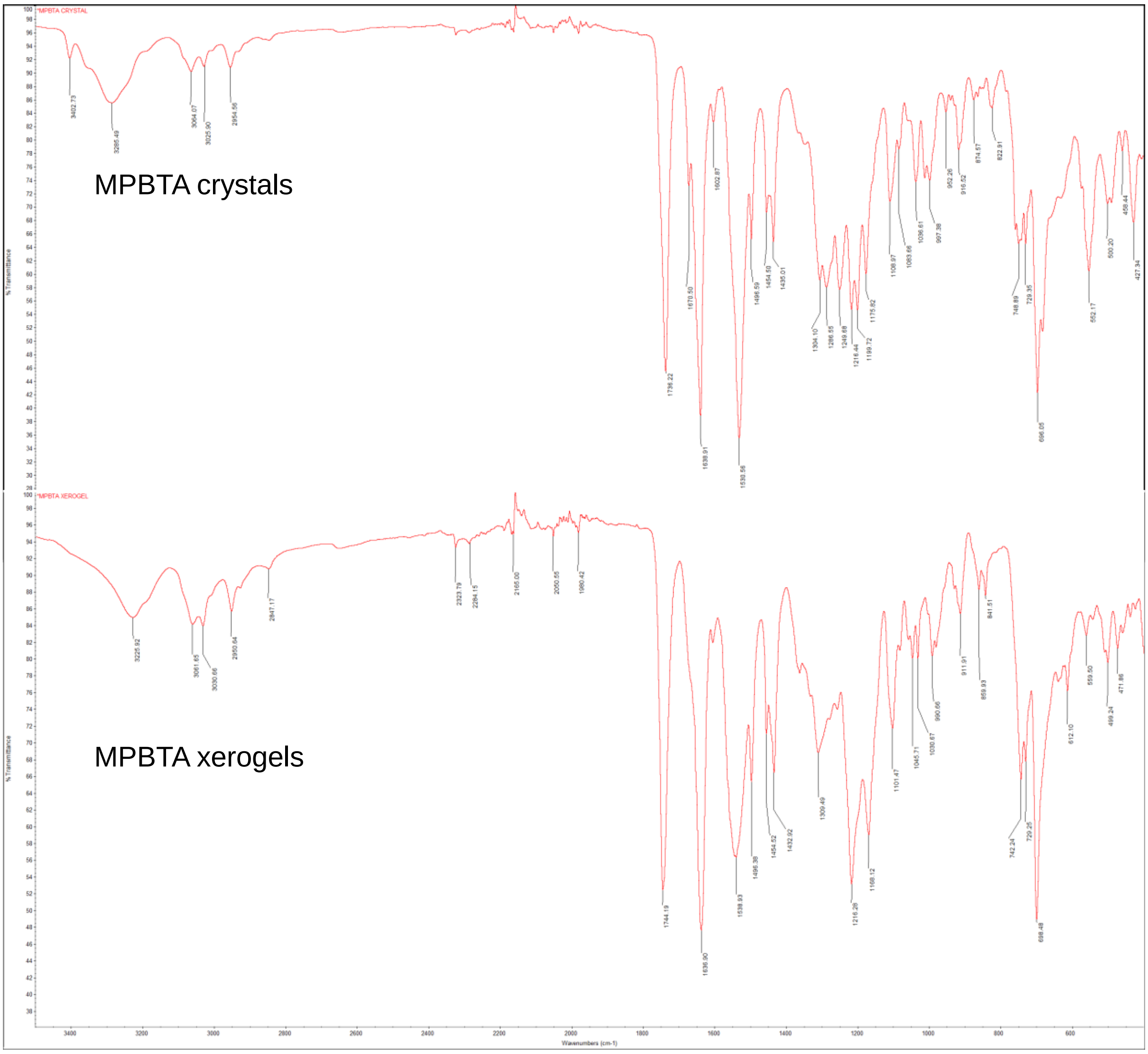

Figure S7: IR spectra of MPBTA crystals (top) and xerogel (bottom) from DMF/H_2O (1:1, v/v) at 2.0 wt/v%.

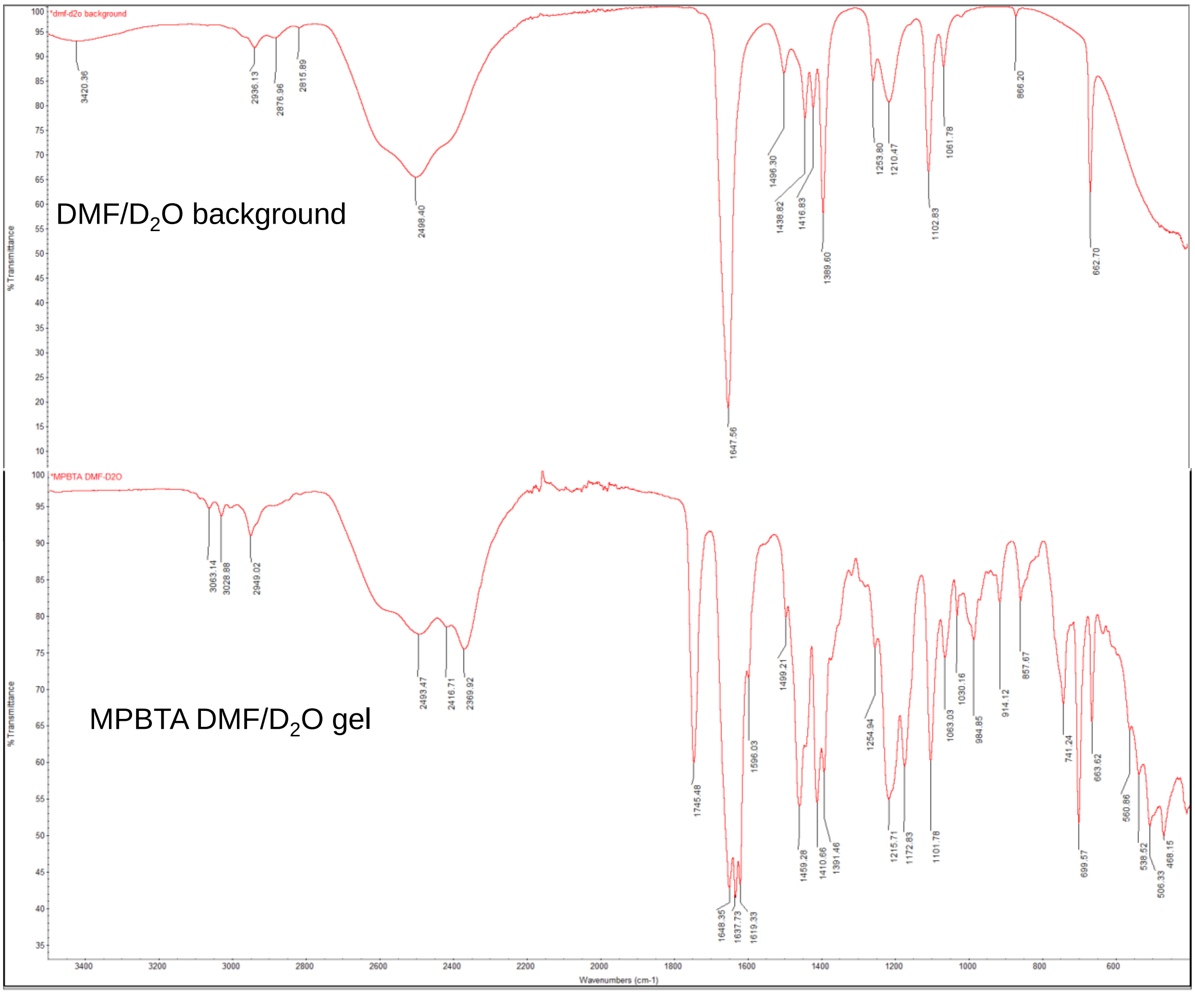

Figure S8: IR spectra of MPBTA gel (bottom) in DMF/D_2O (1:1, v/v) at 2.0 wt/v % and DMF/D_2O (1:1, v/v) mixture (top).

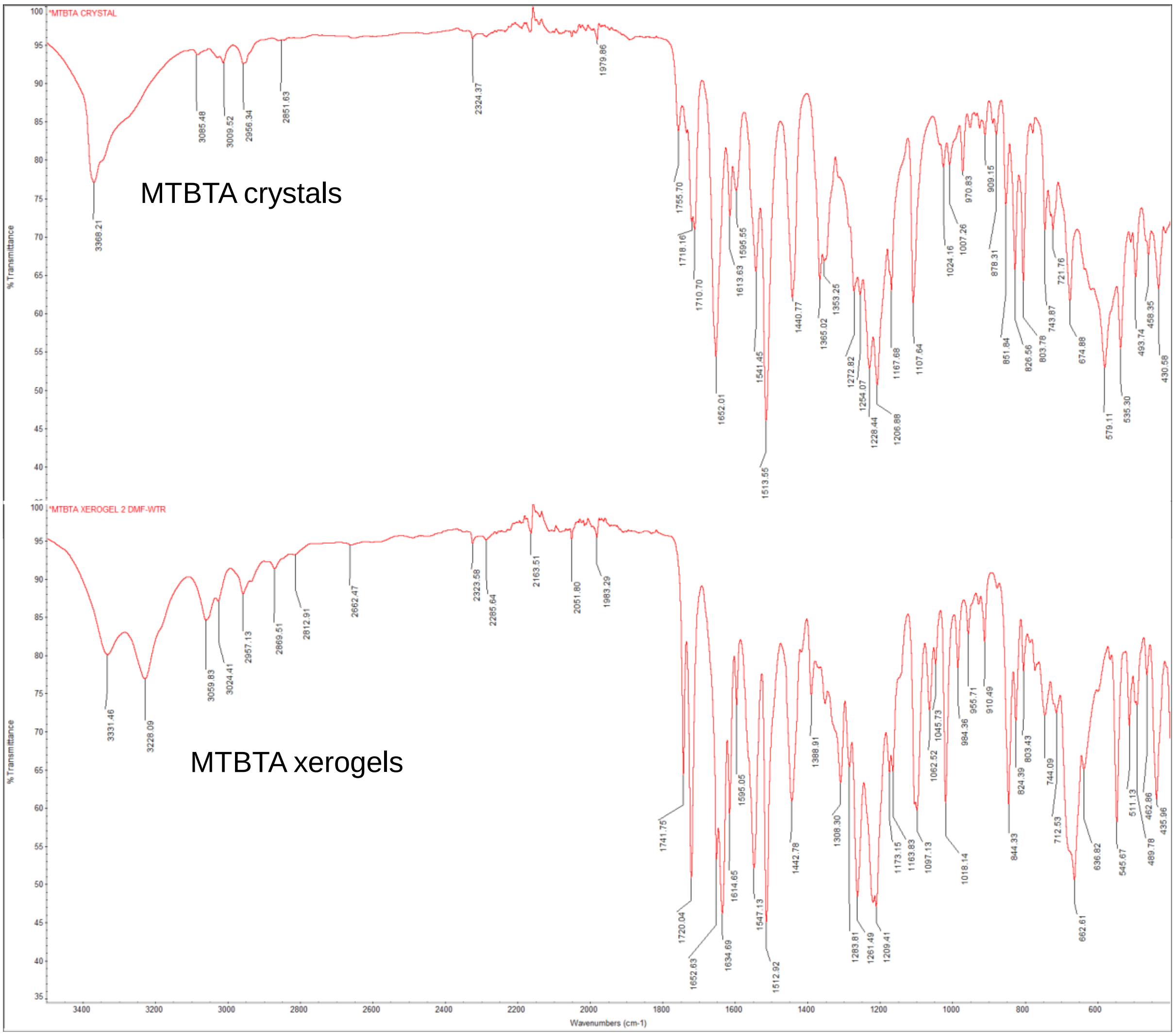

Figure S9: IR spectra of MTBTA: crystal (top) and xerogel (bottom) from DMF/H_2O (1:1, v/v) at 4.5 wt/v %.

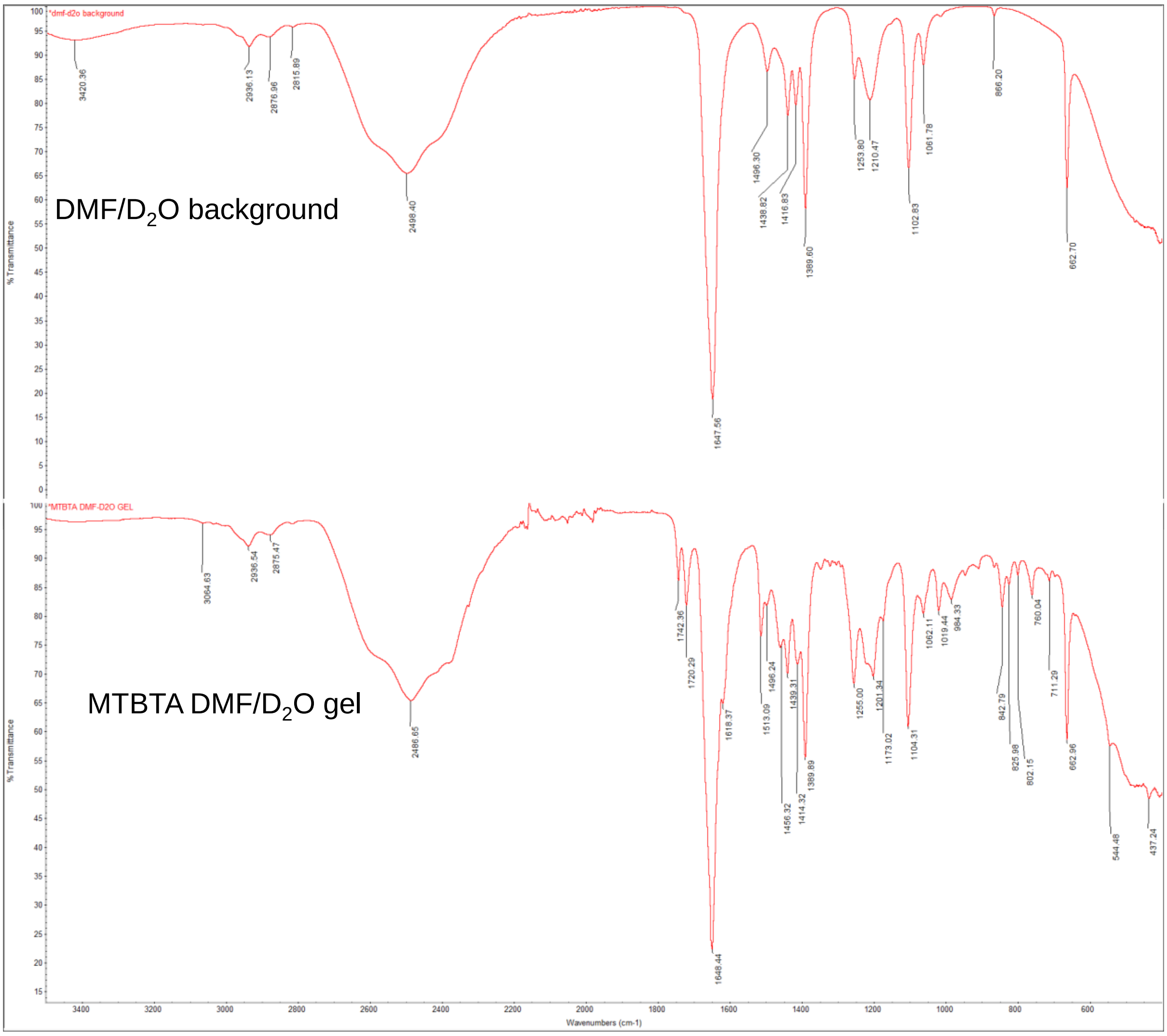

Figure S10: IR spectra of MTBTA gel (bottom) in DMF/D_2O (1:1, v/v) at 4.5 wt/v % and DMF/D_2O (1:1, v/v) mixture (top).

8. Stimuli responsive properties

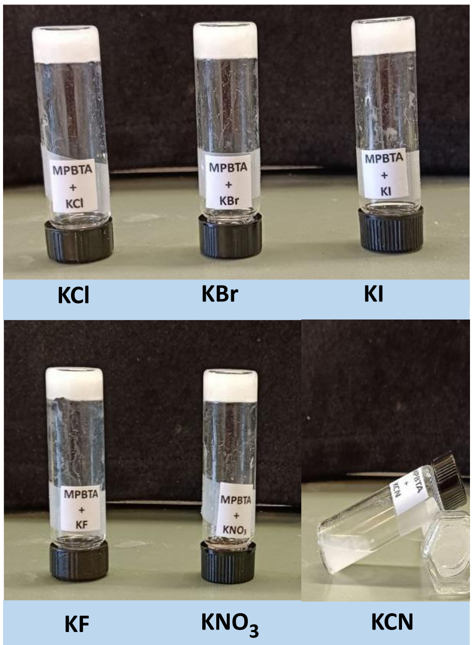

Figure S11. Stimuli-responsive properties of the MPBTA gels at MGC (1.4 wt/v%) in DMF/water mixture (1:1, v/v) with potassium salts.

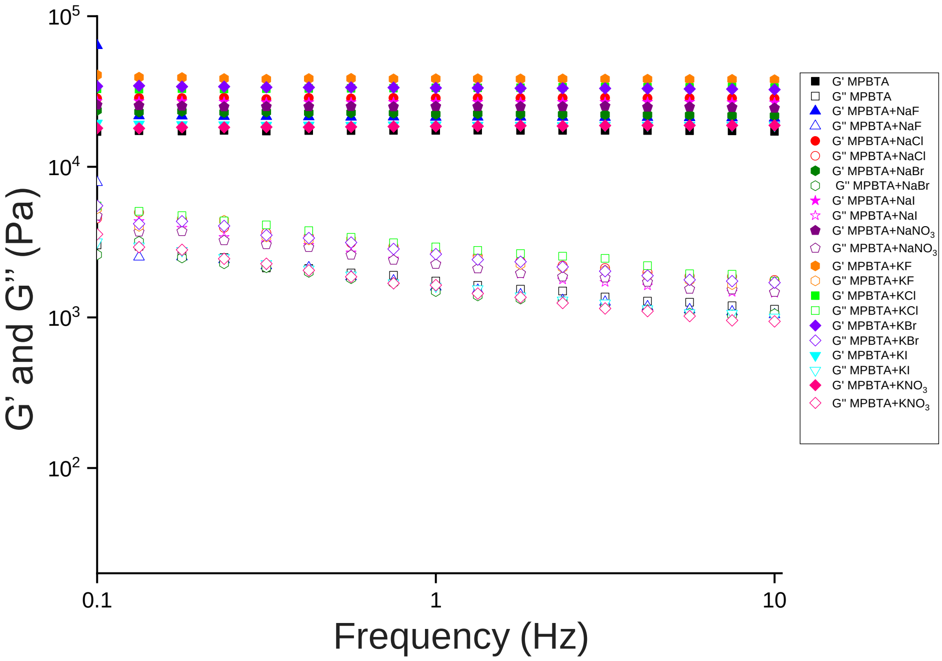

Figure S12. Frequency-sweep experiments of MPBTA gels at MGC (1.4 wt/v%) in the presence of various sodium/potassium salts (1.0 equiv.) of halides and nitrate.

Table S5: T_{gel} studies with MPBTA gels at MGC (1.4 wt/v%) in DMF/water (1:1, v/v), in the presence of 1.0 equivalents of sodium and potassium salts.

Anion	T_{gel} (°C)	
	Without anion (at MGC)	With anion (at MGC)
NaF	74.3	75.8
NaCl	74.3	74.9
NaBr	74.3	75.1
NaI	74.3	74.6
$NaNO_3$	74.3	75.6
KF	74.3	74.6
KCl	74.3	76.7
KBr	74.3	74.5
KI	74.3	74.5
KNO_3	74.3	76.6

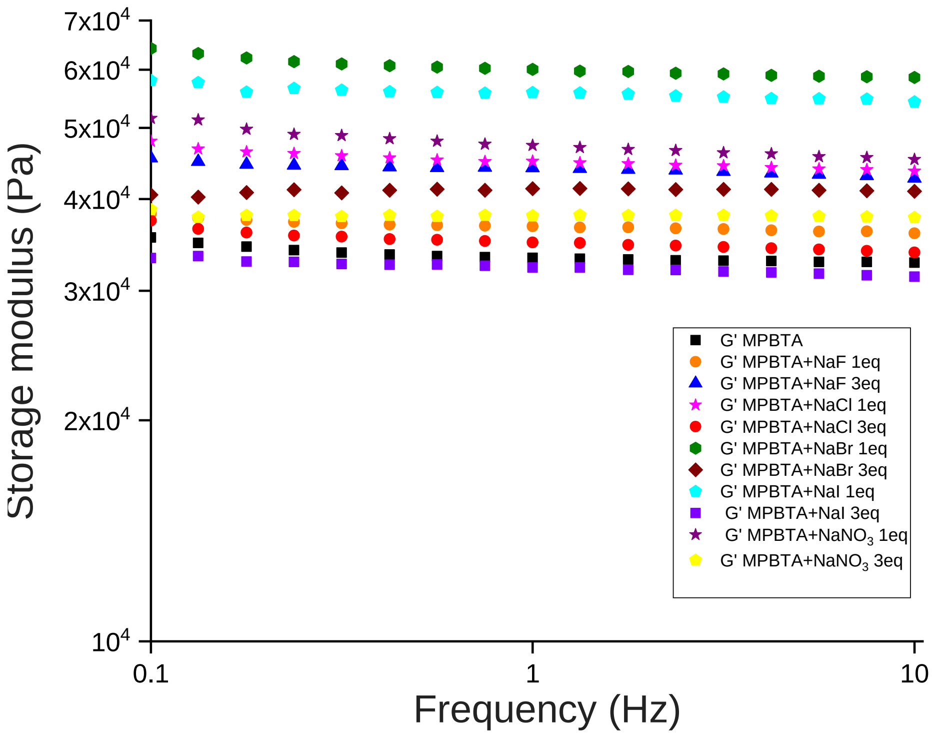

Figure S13. Frequency-sweep experiments of MPBTA gels above MGC (1.8 wt/v%) in the presence of various sodium salts (1.0 equiv. and 3.0 equiv.) of halides and nitrate.

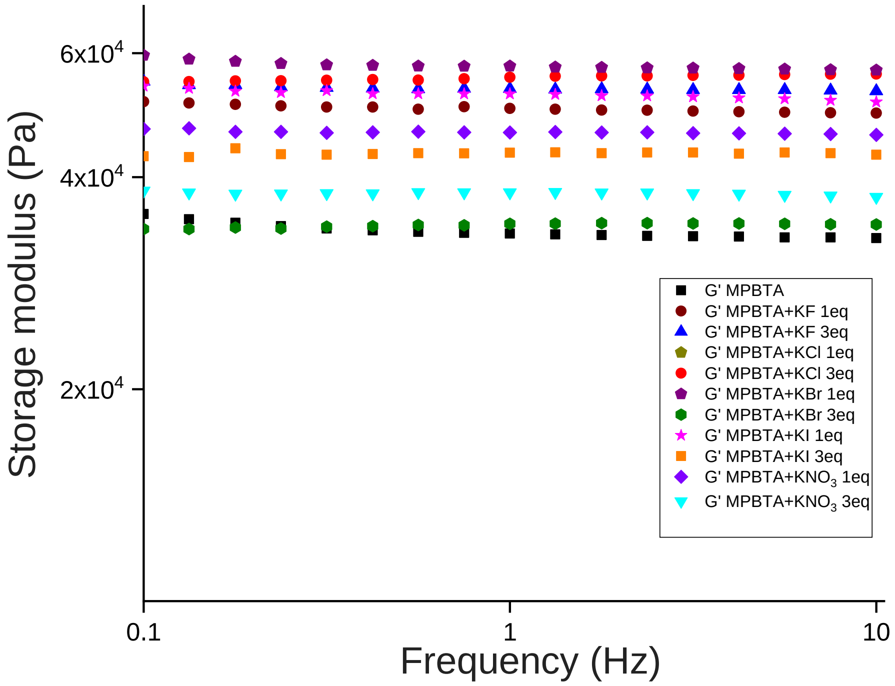

Figure S14. Frequency-sweep experiments of MPBTA gels above MGC (1.8 wt/v%) in the presence of various potassium salts (1.0 equiv. and 3.0 equiv.) of halides and nitrate.

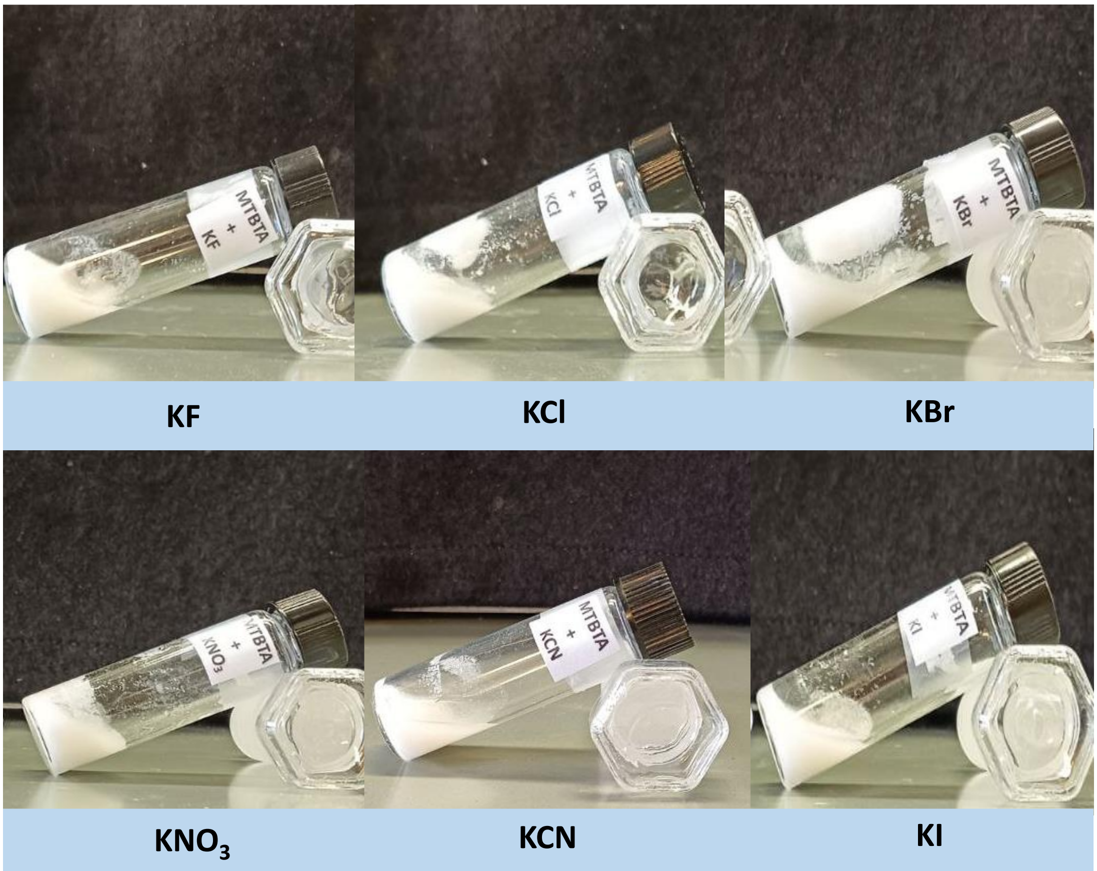

Figure S15. Stimuli-responsive properties of the MTBTA gels in DMF/water mixture (1:1, v/v) with potassium salts (1.0 equiv.).

Table S6: T_{gel} studies with MPBTA gels at MGC (1.4 wt/v%) in DMF/water (1:1, v/v), in the presence of 1.0 equivalents of multi-valent anions of sodium and potassium salts.

Anion	T_{gel} (°C)	
	Without anion (at MGC)	With anion (at MGC)
Na_2SO_4	74.3	76.2
$Na_4P_2O_7$	74.3	75.3
K_2SO_4	74.3	76.4

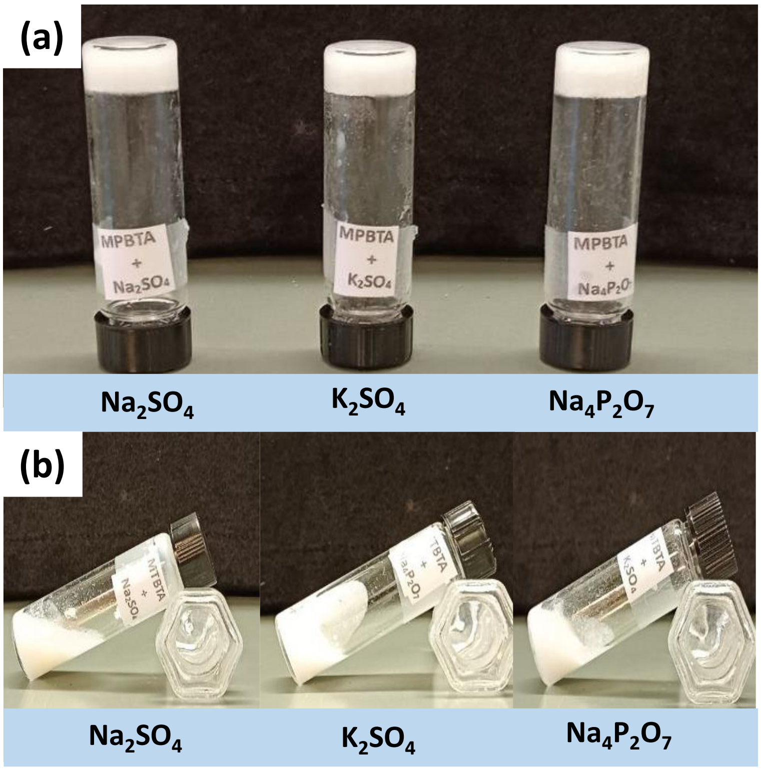

Figure S16. Stimuli-responsive properties of (a) MPBTA gels (1.4 wt/v%) and (b) MTBTA gels (4.5 wt/v%) in DMF/water mixture (1:1, v/v) towards multi-valent anions of sodium and potassium salts.

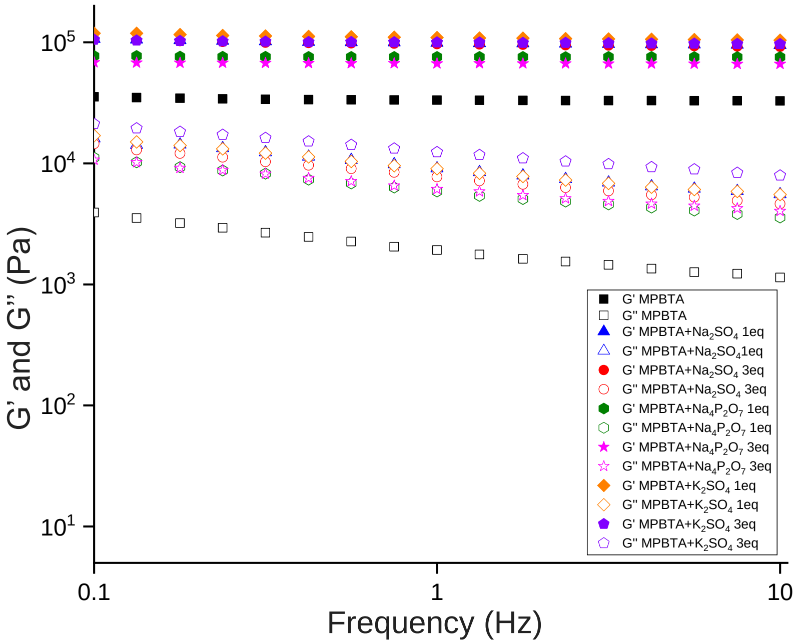

Figure S17. Frequency-sweep experiments of MPBTA gels above MGC (1.8 wt/v%) in the presence of multi-valent anions of sodium and potassium salts (1.0 equiv. and 3.0 equiv.).

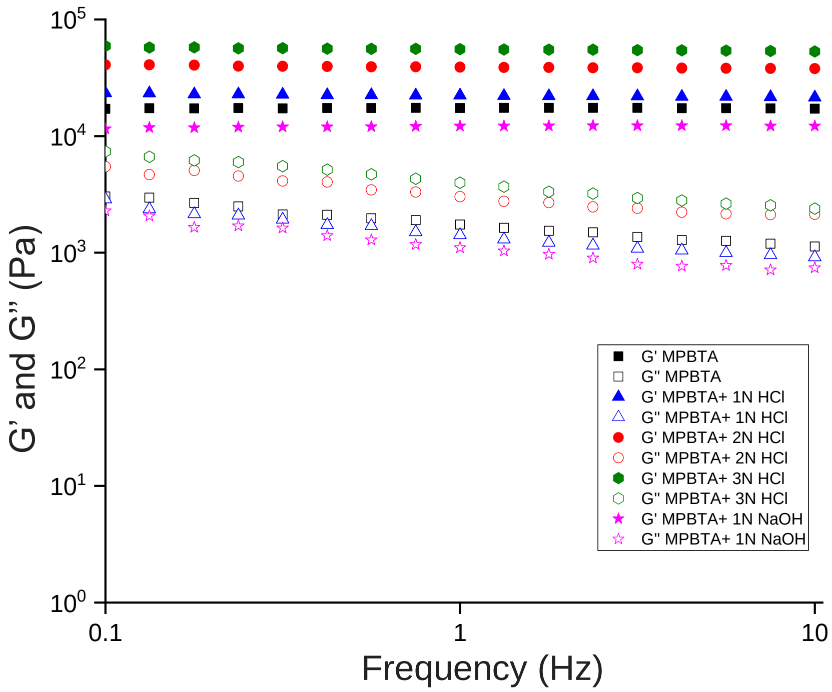

Figure S18. Frequency-sweep experiments of MPBTA gels at MGC (1.4 wt/v%) prepared at different pH.

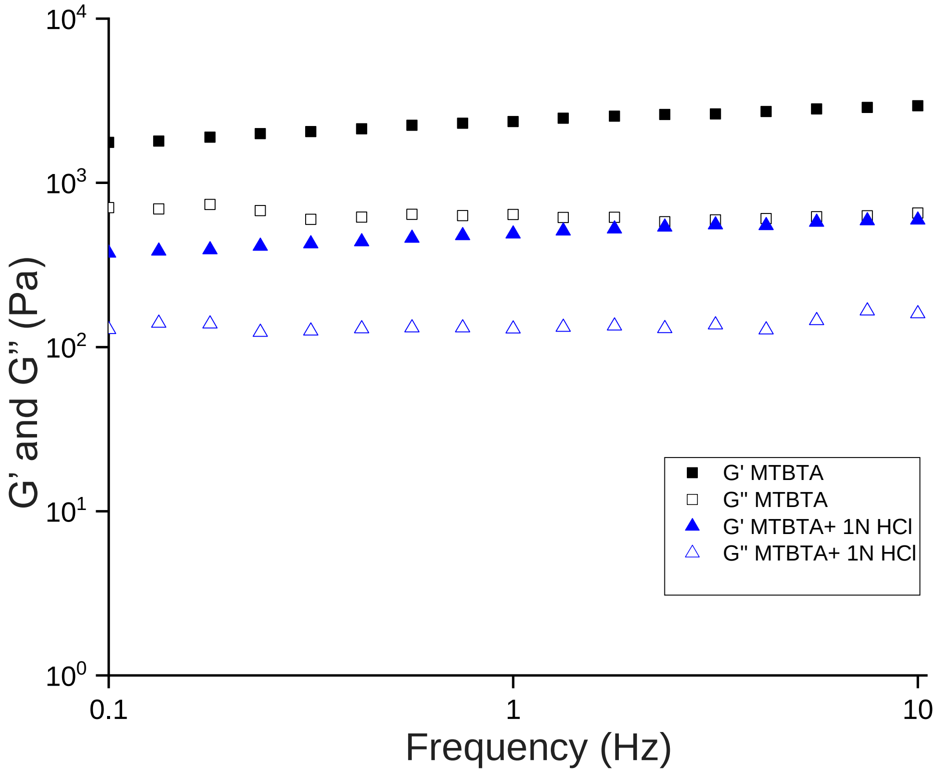

Figure S19. Frequency-sweep experiments of MTBTA gels at MGC (4.5 wt/v%) prepared at different pH.

9. NMR spectra

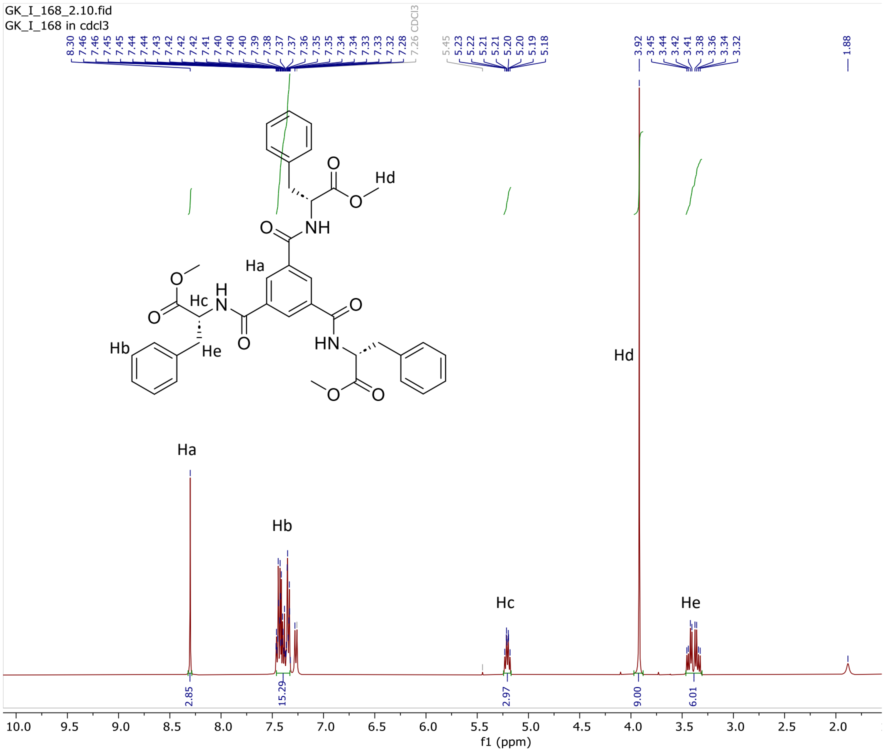

Figure S20. ^{1}H NMR spectrum of MPBTA.

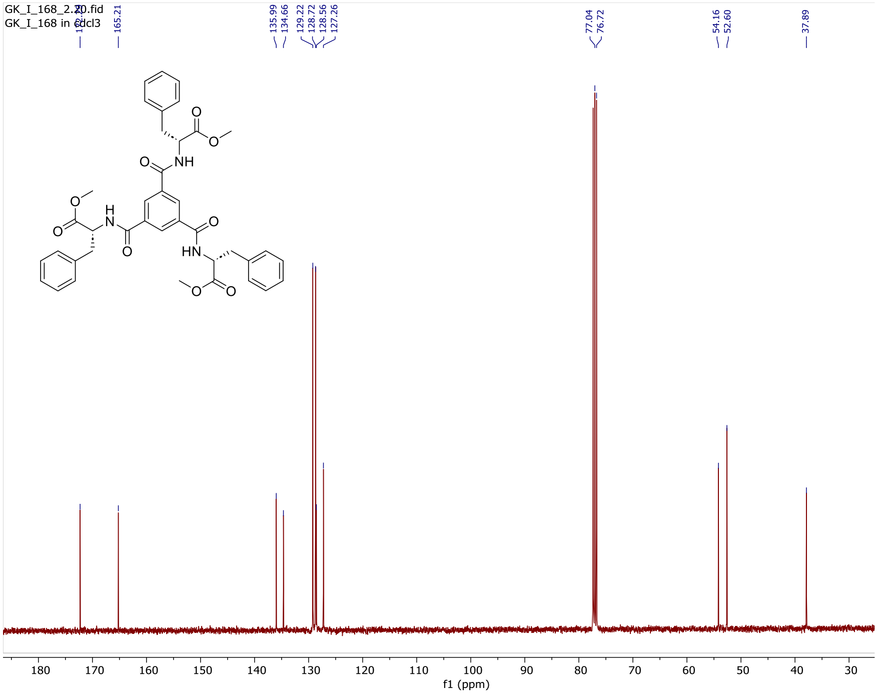

Figure S21. ^{13}C NMR spectrum of MPBTA.

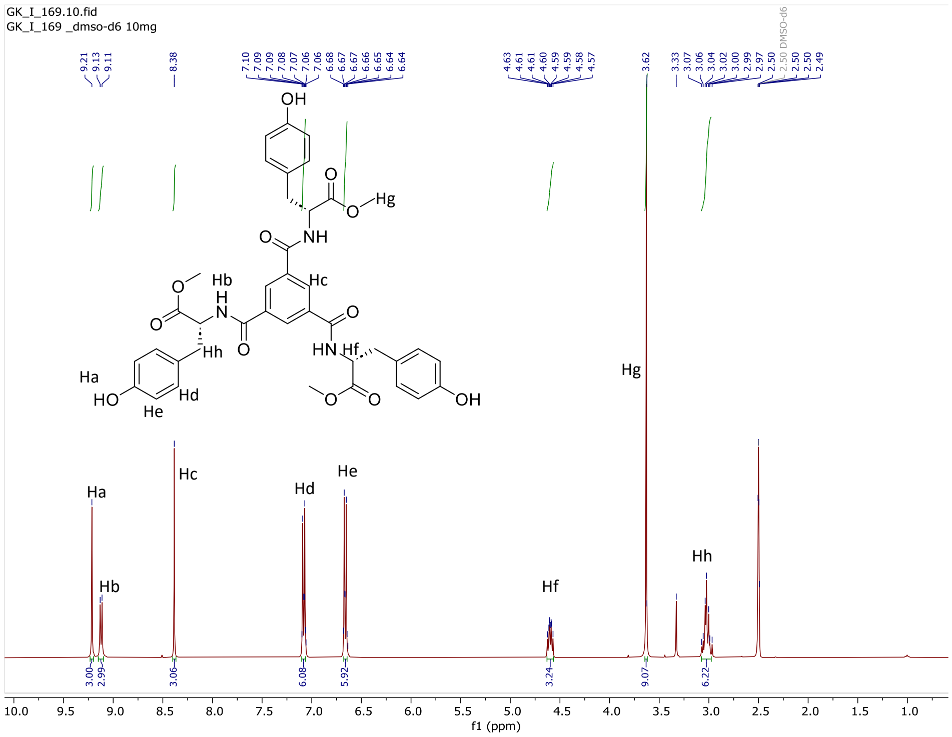

Figure S22. ^{1}H NMR spectrum of MTBTA.

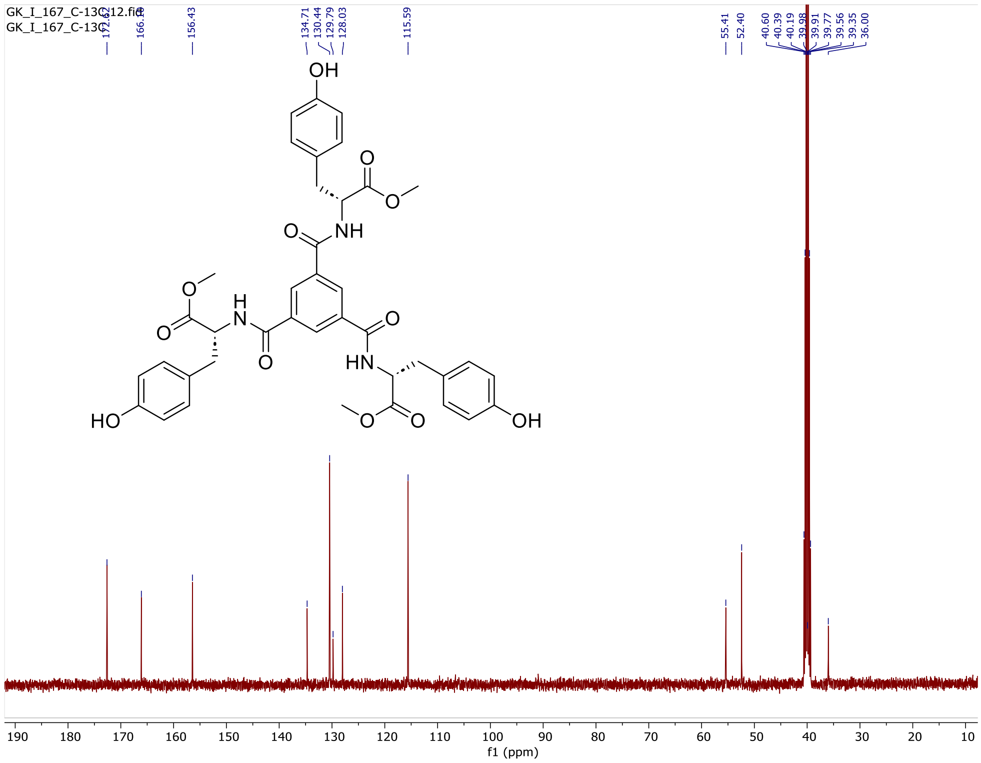

Figure S23. ^{13}C NMR spectrum of MTBTA.

10. Circular dichroism (CD)

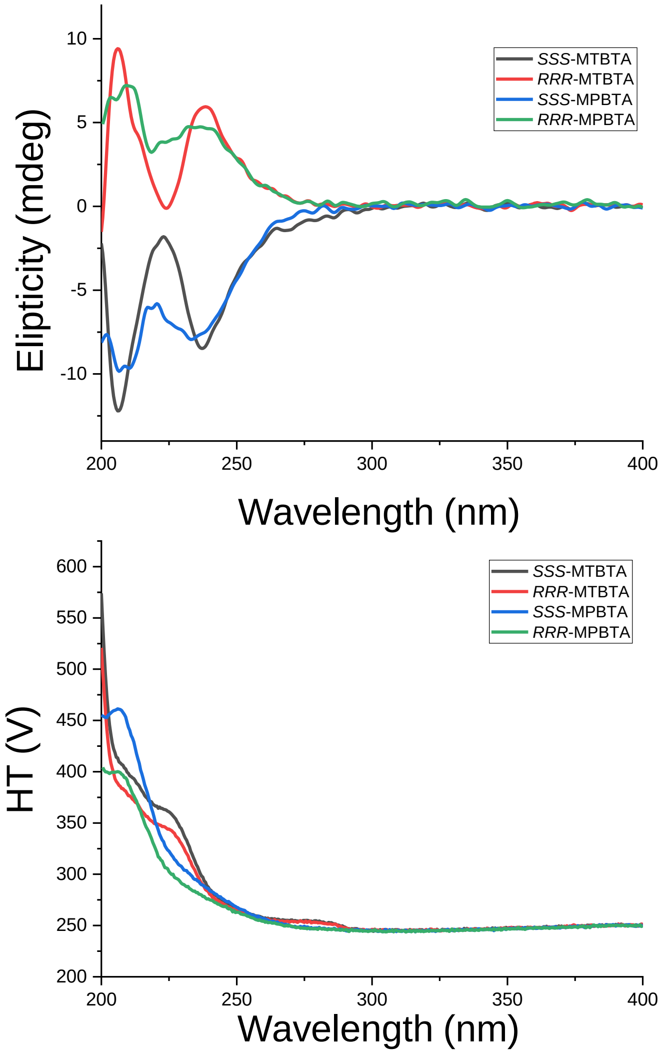

Figure S24. CD spectra for MPBTA and MTBTA compounds (top) and the corresponding HT data (bottom) in the solution state at 0.015 wt/v% in absolute EtOH.
