# Supplementary material for: Designing Stimuli-Responsive Supramolecular Gels by Tuning the Non-Covalent Interactions of the Functional Groups
Source: Gels. 2024 Sep 11;10(9):584. doi: 10.3390/gels10090584 (PMC11430835; doi:10.3390/gels10090584)

checkCIF/PLATON report

Structure factors have been supplied for datablock(s) mo_GK_1_276_1_0m_a

THIS REPORT IS FOR GUIDANCE ONLY. IF USED AS PART OF A REVIEW PROCEDURE FOR PUBLICATION, IT SHOULD NOT REPLACE THE EXPERTISE OF AN EXPERIENCED CRYSTALLOGRAPHIC REFEREE.

No syntax errors found. CIF dictionary Interpreting this report

Datablock: mo_GK_1_276_1_0m_a

```
Bond precision:   C-C = 0.0063 A          Wavelength=0.71073

Cell:             a=9.1493(8)     b=16.3156(13)       c=13.1041(11)
                  alpha=90        beta=106.680(3)     gamma=90
Temperature:      298 K

                Calculated                     Reported
Volume          1873.8(3)                      1873.8(3)
Space group     P 21                           P 21
Hall group      P 2yb                          P 2yb
Moiety formula  C39 H39 N3 O12                 ?
Sum formula     C39 H39 N3 O12                 C39 H39 N3 O12
Mr              741.73                         741.73
Dx,g cm-3       1.315                          1.315
Z               2                              2
Mu (mm-1)       0.098                          0.098
F000            780.0                          780.0
F000'           780.44
h,k,lmax        11,20,16                       11,20,16
Nref            7491[ 3885]                    7428
Tmin,Tmax       0.991,0.996                    0.632,0.745
Tmin'           0.973

Correction method= # Reported T Limits: Tmin=0.632 Tmax=0.745
AbsCorr = MULTI-SCAN

Data completeness= 1.91/0.99       Theta(max)= 26.167

                                                     wR2(reflections)=
R(reflections)= 0.0487( 5037)                        0.1041( 7428)

S = 1.041                   Npar= 511
```

The following ALERTS were generated. Each ALERT has the format
test-name_ALERT_alert-type_alert-level.
Click on the hyperlinks for more details of the test.

Alert level B

PLAT230_ALERT_2_B Hirshfeld Test Diff for O36 --C34 . 12.5 s.u.

Author Response: This is probaly due to disorderd around the ester moiety

PLAT241_ALERT_2_B High 'MainMol' Ueq as Compared to Neighbors of O36 Check

Author Response: This is probaly due to disorderd around the ester moiety

Alert level C

STRVA01_ALERT_4_C Flack parameter is too small
From the CIF: _refine_ls_abs_structure_Flack -0.300
From the CIF: _refine_ls_abs_structure_Flack_su 0.400

Author Response: This is because the compound may be a weak anomalous scatterer

PLAT213_ALERT_2_C Atom O36 has ADP max/min Ratio ..... 3.4 prolat

Author Response: This is probaly due to disorderd around the ester moiety

PLAT220_ALERT_2_C NonSolvent Resd 1 C Ueq(max)/Ueq(min) Range 4.5 Ratio

Author Response: The assignment of atoms were correct

PLAT220_ALERT_2_C NonSolvent Resd 1 O Ueq(max)/Ueq(min) Range 3.7 Ratio

Author Response: The assignment of atoms were correct

PLAT222_ALERT_3_C NonSolvent Resd 1 H Uiso(max)/Uiso(min) Range 5.2 Ratio

Author Response: The assignment of atoms were correct

PLAT241_ALERT_2_C High 'MainMol' Ueq as Compared to Neighbors of C48 Check

Author Response: This is probaly due to disorderd around the ester moiety

PLAT242_ALERT_2_C Low 'MainMol' Ueq as Compared to Neighbors of C43 Check

Author Response: The assignment of carbon atom is correct and this alert may be due to the disorder in the structure

PLAT242_ALERT_2_C Low 'MainMol' Ueq as Compared to Neighbors of C46 Check

Author Response: The assignment of carbon atom is correct and this alert may be due to the disorder in the structure

PLAT334_ALERT_2_C Small <C-C> Benzene Dist. C43 -C49 . 1.37 Ang.

Author Response: This is probaly due to disorder in the molecule

PLAT340_ALERT_3_C Low Bond Precision on C-C Bonds .... 0.00633 Ang.

Author Response: This is probaly due to disorder in the molecule

PLAT910_ALERT_3_C Missing # of FCF Reflection(s) Below Theta(Min). 7 Note
1 0 0, 1 1 0, 0 2 0, -1 0 1, 0 0 1, -1 1 1,
0 1 1,

Author Response: These low angle reflections are probably missing due to the beamstop.

Alert level G

PLAT007_ALERT_5_G Number of Unrefined Donor-H Atoms .... 6 Report
H9 H16 H25 H31 H40 H47

PLAT032_ALERT_4_G Std. Uncertainty on Flack Parameter Value High . 0.400 Report

PLAT230_ALERT_2_G Hirshfeld Test Diff for O35 --C34 . 7.0 s.u.

Author Response: This is probaly due to disorderd around the ester moiety

PLAT230_ALERT_2_G Hirshfeld Test Diff for O36 --O35A . 10.0 s.u.

Author Response: This is probaly due to disorderd around the ester moiety

PLAT301_ALERT_3_G Main Residue Disorder ....(Resd 1) 4% Note

PLAT395_ALERT_2_G Deviating X-O-Y Angle From 120 for O36 . 52.6 Degree

PLAT720_ALERT_4_G Number of Unusual/Non-Standard Labels .... 2 Note
C018 H018

PLAT721_ALERT_1_G Bond Calc 0.97000, Rep 0.96000 Dev... 0.01 Ang.
C37A -H37D 1_555 1_555 .... # 72 Check

PLAT791_ALERT_4_G Model has Chirality at C10 (Sohncke SpGr) R Verify

PLAT791_ALERT_4_G Model has Chirality at C26 (Sohncke SpGr) R Verify

PLAT791_ALERT_4_G Model has Chirality at C41 (Sohncke SpGr) R Verify

```
PLAT912_ALERT_4_G Missing # of FCF Reflections Above STh/L=  0.600         18 Note
PLAT969_ALERT_5_G The 'Henn et al.' R-Factor-gap value ..........      3.325 Note
              Predicted wR2: Based on SigI**2  3.13 or SHELX Weight 10.00
PLAT978_ALERT_2_G Number C-C Bonds with Positive Residual Density.          0 Info
```

```
   0 ALERT level A = Most likely a serious problem - resolve or explain
   2 ALERT level B = A potentially serious problem, consider carefully
  11 ALERT level C = Check. Ensure it is not caused by an omission or oversight
  14 ALERT level G = General information/check it is not something unexpected

   1 ALERT type 1 CIF construction/syntax error, inconsistent or missing data
  13 ALERT type 2 Indicator that the structure model may be wrong or deficient
   4 ALERT type 3 Indicator that the structure quality may be low
   7 ALERT type 4 Improvement, methodology, query or suggestion
   2 ALERT type 5 Informative message, check
```

It is advisable to attempt to resolve as many as possible of the alerts in all categories. Often the minor alerts point to easily fixed oversights, errors and omissions in your CIF or refinement strategy, so attention to these fine details can be worthwhile. In order to resolve some of the more serious problems it may be necessary to carry out additional measurements or structure refinements. However, the purpose of your study may justify the reported deviations and the more serious of these should normally be commented upon in the discussion or experimental section of a paper or in the "special_details" fields of the CIF. checkCIF was carefully designed to identify outliers and unusual parameters, but every test has its limitations and alerts that are not important in a particular case may appear. Conversely, the absence of alerts does not guarantee there are no aspects of the results needing attention. It is up to the individual to critically assess their own results and, if necessary, seek expert advice.

Publication of your CIF in IUCr journals

A basic structural check has been run on your CIF. These basic checks will be run on all CIFs submitted for publication in IUCr journals (*Acta Crystallographica*, *Journal of Applied Crystallography*, *Journal of Synchrotron Radiation*); however, if you intend to submit to *Acta Crystallographica Section C* or *E* or *IUCrData*, you should make sure that full publication checks are run on the final version of your CIF prior to submission.

Publication of your CIF in other journals

Please refer to the *Notes for Authors* of the relevant journal for any special instructions relating to CIF submission.

PLATON version of 15/07/2024; check.def file version of 15/07/2024

Datablock mo_GK_1_276_1_0m_a - ellipsoid plot

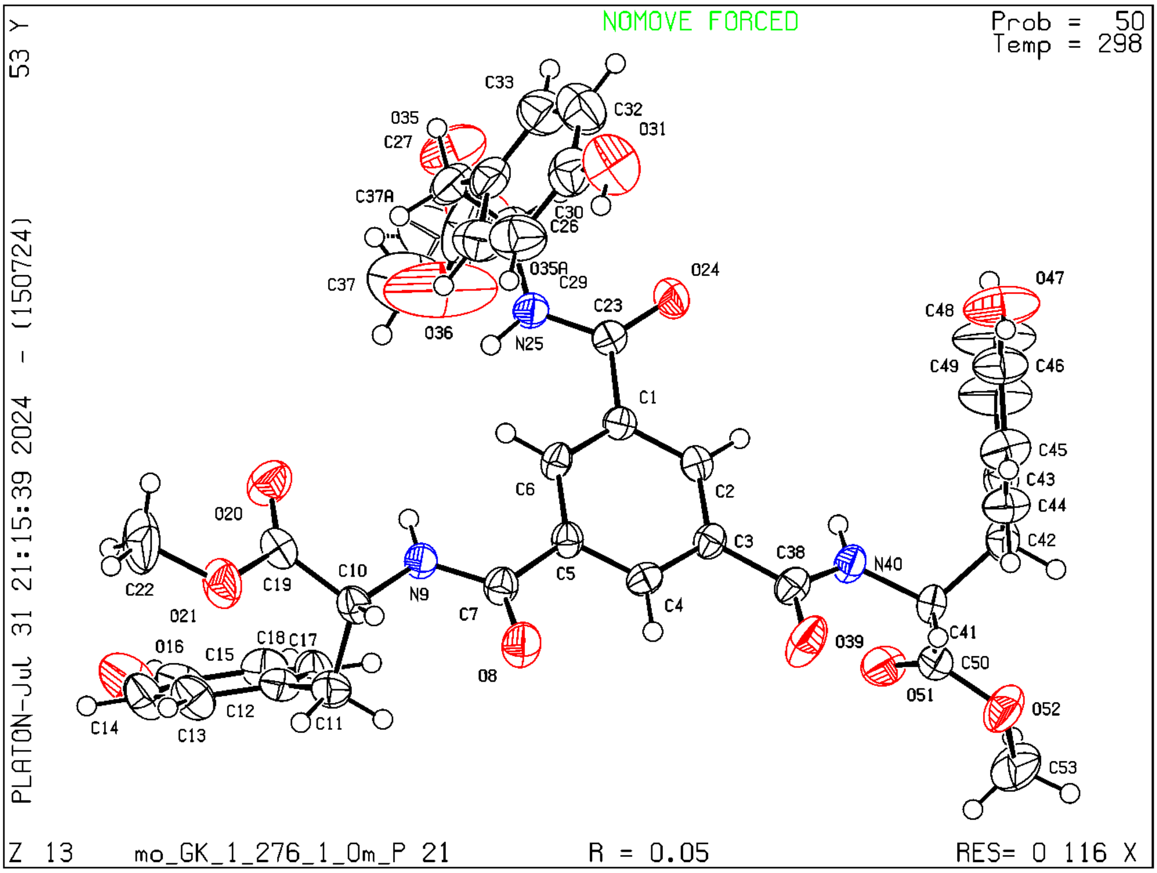

Supplement: Supplementary file 1 [file checkCIF.pdf › Supplementary Materials/checkCIF.pdf]
